# Compost fermented with thermophilic *Bacillaceae* reduces heat stress–induced mortality in laying hens through gut microbial modulation

**DOI:** 10.1186/s42523-026-00520-5

**Published:** 2026-02-03

**Authors:** Yudai Inabu, Hirokuni Miyamoto, Hideyuki Takahashi, Tamotsu Kato, Shigeharu Moriya, Atsushi Kurotani, Haruki Yamano, Teruno Nakaguma, Naoko Tsuji, Chitose Ishii, Makiko Matsuura, Satoshi Wada, Takashi Satoh, Motoaki Udagawa, Hisashi Miyamoto, Jun Kikuchi, Hiroaki Kodama, Hiroshi Ohno

**Affiliations:** 1 Kuju Agricultural Research Center, Graduate School of Agriculture, Kyushu University, Oita, 878-0201 Japan; 2 Graduate School of Integrated Sciences for Life, Hiroshima University, Hiroshima, Japan; 3 Graduate School of Horticulture, Chiba University, Matsudo, 271‑8501 Japan; 4 Graduate School of Medical Life Science, Yokohama City University, Tsurumi, Yokohama 230-0045 Japan; 5 RIKEN Center for Integrative Medical Sciences, Yokohama, Kanagawa, 230-0045 Japan; 6 Japan Eco-Science (Nikkan Kagaku) Co., Ltd., Chiba, 260-0034 Japan; 7 Sermas, Co., Ltd., Chiba, 271-8501 Japan; 8 RIKEN, Center for Advanced Photonics, Wako, Saitama, 351-0198 Japan; 9 Research Center for Agricultural Information Technology, National Agriculture and Food Research Organization, Tsukuba, Ibaraki, 305-0856 Japan; 10 Division of Hematology, Kitasato University School of Allied Health Sciences, Sagamihara, Kanagawa, 252-0373 Japan; 11 Keiyo Gas Energy Solution Co., Ltd., Ichikawa, Chiba, 272-0033 Japan; 12 Miroku Co., Ltd., Kitsuki, Oita, 873-0021 Japan; 13 RIKEN Center for Sustainable Resource Science, Yokohama, Kanagawa, 230-0045 Japan

**Keywords:** Faecal microbiota, Thermophile, Heat stress

## Abstract

**Background:**

Heat stress (HS) adversely affects poultry health and productivity. Recently, it has been suggested that the gut microbiota may play a role in host resilience to HS, although the details of its mechanism remain unclear. Here, the heat tolerance-related effects of dietary supplementation of compost fermented by the thermophile *Bacillaceae* were explored using a laying hen model (601,474 hens in total).

**Results:**

In a field study conducted during the summer (maximum temperatures of approximately 35 °C) in eleven hen houses, oral administration of the compost extract resulted in a statistically significant reduction in mortality. Difference-in-differences analysis revealed that the abundances of the genera *Lachnospiraceae* NK3A20 group, *Enterococcus*, *Ruminococcus* 2, *Blautia*, *Lactobacillus*, *Christensenellaceae* R-7 group, and *Tyzzerella* 4 were significantly increased by compost administration, whereas those of the *Prevotellaceae* NK3B31 group, *Prevotella* 9, *Romboutsia*, *Turicibacter*, and *Escherichia–Shigella* were significantly reduced. In addition, to evaluate the relationship between short-chain fatty acids (SCFAs) metabolic profiles and the gut bacterial population, factor analysis combined with feature selection based on multiple machine learning (ML) algorithms was performed. The resulting optimal structural equation model suggested that compost administration led to increases in the levels of the SCFAs acetate and butyrate, as well as decreases in the levels of the genera *Romboutsia* and *Turicibacter*.

**Conclusion:**

Oral administration of thermophile-fermented compost to laying hens alleviated HS-induced mortality. Integrative computational evaluations further revealed that the reduction in mortality was linked to structural changes in the gut microbiota composition and SCFA concentrations.

**Supplementary Information:**

The online version contains supplementary material available at 10.1186/s42523-026-00520-5.

## Background

Heat stress (HS) presents problems for livestock production; many heat waves have caused devastating economic losses as a result of increased mortality. In particular, birds are more sensitive to high temperatures than are other monogastric animals because of feather coverage and the absence of sweat glands [1]. Therefore, HS is among the most critical environmental factors that might negatively impact poultry production and health [2]. HS negatively affects mortality and panting rate among laying hens [3]. Additionally, HS has been shown to induce significant decreases in production indices, including egg mass and egg yield, among laying hens [4]. Considering these findings, research on the effects of heat stress on chickens and relevant countermeasures is essential.

The microbiota likely plays an important role in stress responses [5, 6], and its composition is associated with heat tolerance [7]. Intestinal microorganisms can contribute to intestinal barrier maintenance, thus effectively ensuring host health [8]. Disruption of the intestinal temperature may enable pathogen invasion and the consequent development of disease [9], thus leading to the need for antibiotics [10]. However, antibiotics overuse and misuse are a worldwide problem that has led to the emergence of antimicrobial-resistant bacteria. The World Health Organization (WHO) launched an action plan against antimicrobial resistance (AMR) [11] that included elimination of the use of antibiotics for growth promotion in livestock by 2015. Additionally, our recent causal research suggested that the use of antibiotics can negatively affect immune system function and may positively affect the generation of methane, a greenhouse gas [12]. Therefore, the development of antimicrobial-independent husbandry techniques is essential for livestock production.

Recent studies have investigated the effects of probiotics and prebiotics on the adverse effects of HS among livestock. Probiotics and prebiotics can ameliorate the adverse effects on intestinal and liver circulation caused by heat stress [13, 14]. For example, studies have reported that intestinal barrier function in broilers can be improved by *Bacillus subtilis* alone [15] or in combination with lactic acid bacteria [16]. Our previous studies revealed that oral administration of compost containing thermophilic *Bacillaceae* improved fecundity and quality by modifying the intestinal bacterial population in flatfish, chickens, and pigs [17–22]. On hen farms, the administration of compost fermented with marine animal resources has been shown to improve egg production in hens [22]. However, whether feeding thermophile-fermented compost to laying hens mitigates the adverse effects of HS is unclear. Therefore, the aim of this study was to elucidate the effects of feeding compost extract to egg-laying hens on mortality and faecal environmental conditions under HS. Specifically, the mortality, faecal bacterial diversity, and organic acid contents of egg-laying hens that were fed compost under HS during the summer were compared with those of a nontreated group in the autumn (normal temperatures). Furthermore, the relationships of the selected components were computationally inferred via machine learning (ML) algorithms, exploratory factor analysis (EFA) and structural equation modelling (SEM) as statistical factor analyses and causal inference (CI). Elucidating these relationships may provide insight into important gut metabolic structures for heat control in livestock animals.

## Results

### Validation of the mortality rates of hens in heat-stressed environments

**Fig. 1:**
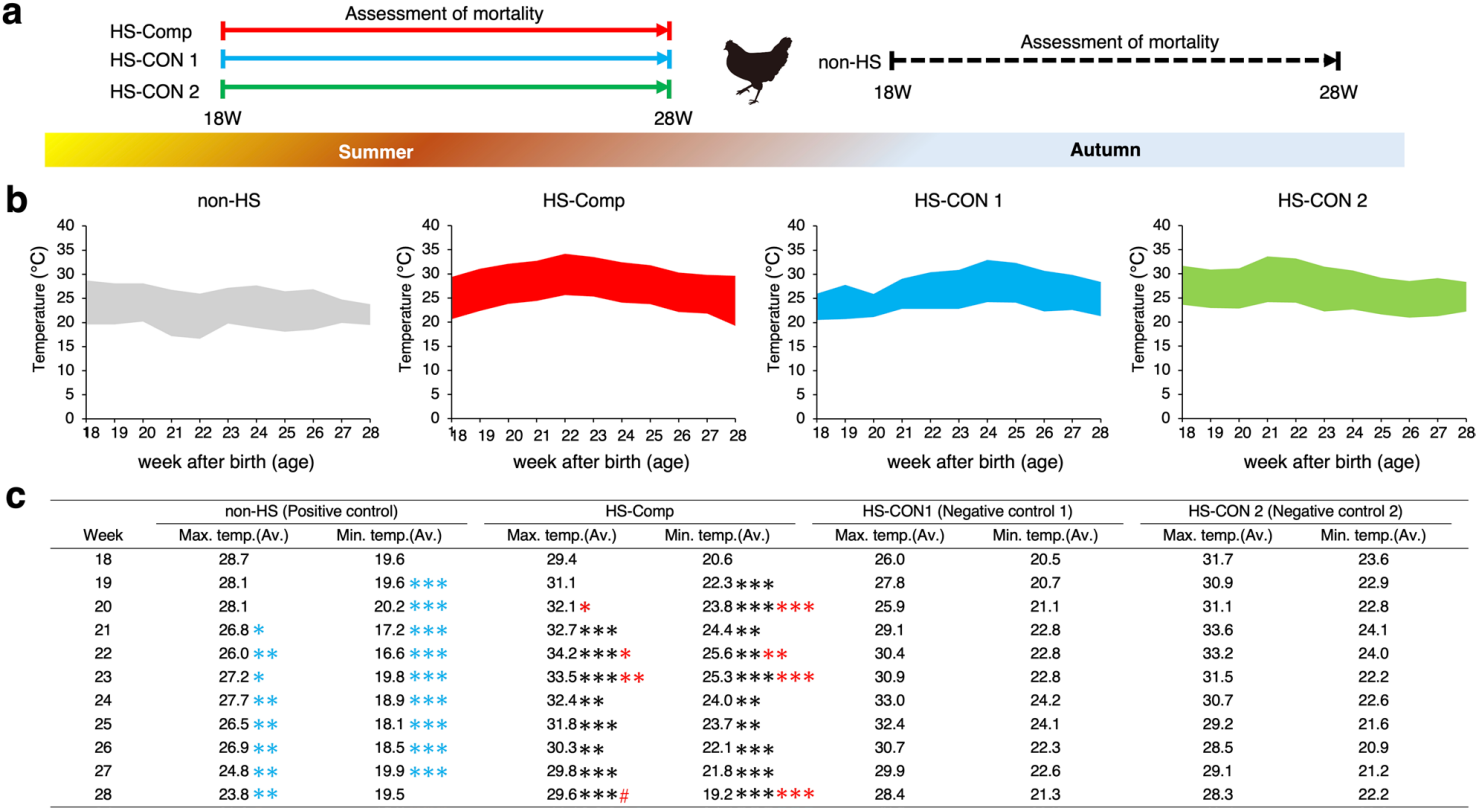
The temperatures of the hen farms during the period of rearing. (**a**) Comparison of farm conditions. The mortality rates of chickens between 18 and 28 weeks after birth were compared. (**b**) Hen house temperature profiles and (**c**) corresponding raw data during the rearing period. The “max_temp” and “min_temp” indicate the average maximum and minimum temperatures, respectively. The data of each hen house are listed according to the following criteria. The “non-HS” indicates the data from the rearing period in early autumn. Three groups were established according to the compost application conditions in July and August of 2015. The “HS-compost” indicates the group that was administered the compost-derived thermophile extract. The “HS-CON1” and “HS-CON2” indicate the groups not administered compost. The blue, black, and red asterisks indicate non-HS vs. HS-CON, non-HS vs. HS-compost (HS-comp), and HS-compost and HS-CON, respectively. Significance is indicated as follows: #, *p* < 0.2; *, *p* < 0.05; **, *p* < 0.01; and ***, *p* < 0.001

**Fig. 2:**
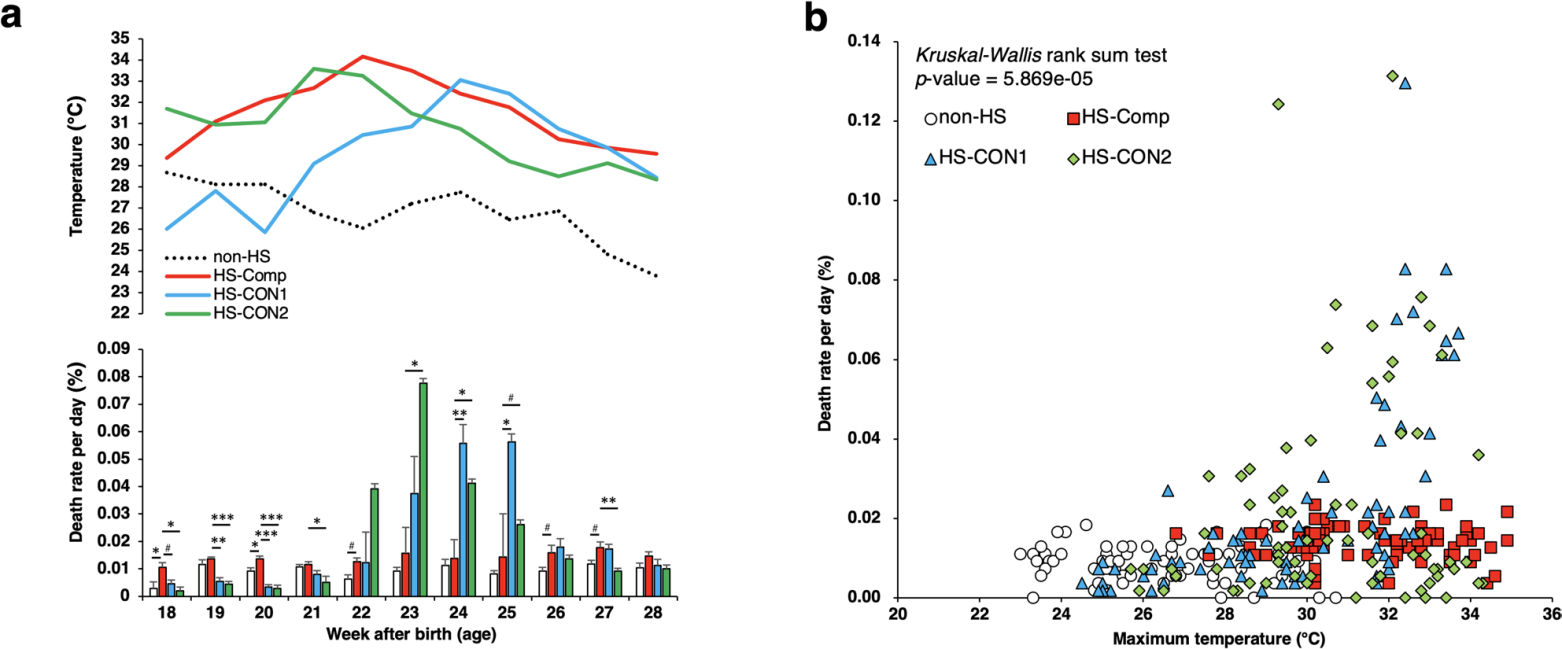
Relationships between temperature and mortality in chickens. (**a**) Relationships between the mortality rate and temperature at each week of age. The “Week after birth (age)” indicates age after birth. The profiles of Max_temp in Figs. 1b and 1c are shown in the upper portion of the figure. (**b**) Mortality rates and maximum temperatures of the tested farms during the period of rearing. The white circle, red square, blue triangle, and green lozenge represent the non-heat stress (non-HS), HS-Comp (HS-compost), HS-CON1, and HS-CON2 groups, respectively. The abbreviations used were as follows: non-HS, moderate-temperature data (autumn); HS-CON1 and HS-CON2, data of hen houses under high temperatures (July and August); and HS-Comp, the data of a hen house administered the compost extract under high temperatures (July and August); #, *p* < 0.2; *, *p* < 0.05; **, *p* < 0.01; and ***, *p* < 0.001

Four groups of 120-day-old laying hens were established: one non-heat-stressed group (non-HS) reared in autumn and three heat-stressed groups (HS-Comp, HS-CON1, and HS-CON2) reared in the summer (Fig. 1a). Only the HS-Comp group received compost extract. Because each farm housed a different commercial layer strain, we cannot exclude the possibility that breed differences contributed to the observed variation. Therefore, the non-HS group was used as a reference for seasonal effects, whereas the HS-CON2 group provided an additional comparison with another strain under HS to help contextualize the treatment effects across strains. For the non-HS group, the average maximum and minimum temperatures ranged from 23.8 to 28.7 °C and from 16.6 to 20.2 °C, respectively (Fig. 1b and c, and 2a). In the HS-Comp, HS-CON 1, and HS-CON 2 groups, the average maximum temperatures ranged from 29.4 ~ 34.2 °C, 25.9 ~ 33.0 °C, and 28.3 ~ 33.6 °C, respectively, whereas the average minimum temperatures ranged from 19.2 ~ 25.6 °C, 20.5 ~ 24.1 °C, and 20.9 ~ 24.1 °C, respectively (Fig. 1b and c, and 2a). The ambient temperature was higher in the HS groups than in the non-HS group (*p* < 0.05). The mortality rates per week of age are shown in Fig. 2a, and the relationship between mortality and maximum ambient temperature is illustrated in Fig. 2b. The mortality rate for the non-HS group was less than 0.02% throughout the study period. Similarly, the mortality rate in the HS-Comp group rarely exceeded 0.02% and remained below 0.03% at all weeks of age. At 18 and 20 weeks of age, the mortality rate in the HS-Comp group was greater than that in the non-HS group (*p* < 0.05), likely because of HS; however, no significant differences in mortality were observed between the two groups after 21 weeks of age. In contrast, the HS-CON groups often presented higher mortality rates than the non-HS group did, particularly from 23 to 25 weeks of age (*p* < 0.05), likely due to HS. As a result, the cumulative number of deaths in the HS-CON groups tended to increase after 23 weeks of age compared with that in the non-HS and HS-Comp groups (Fig. S1). Therefore, the increased mortality due to HS during the summer was alleviated by the administration of compost extracts containing thermophiles.

**Fig. 3:**
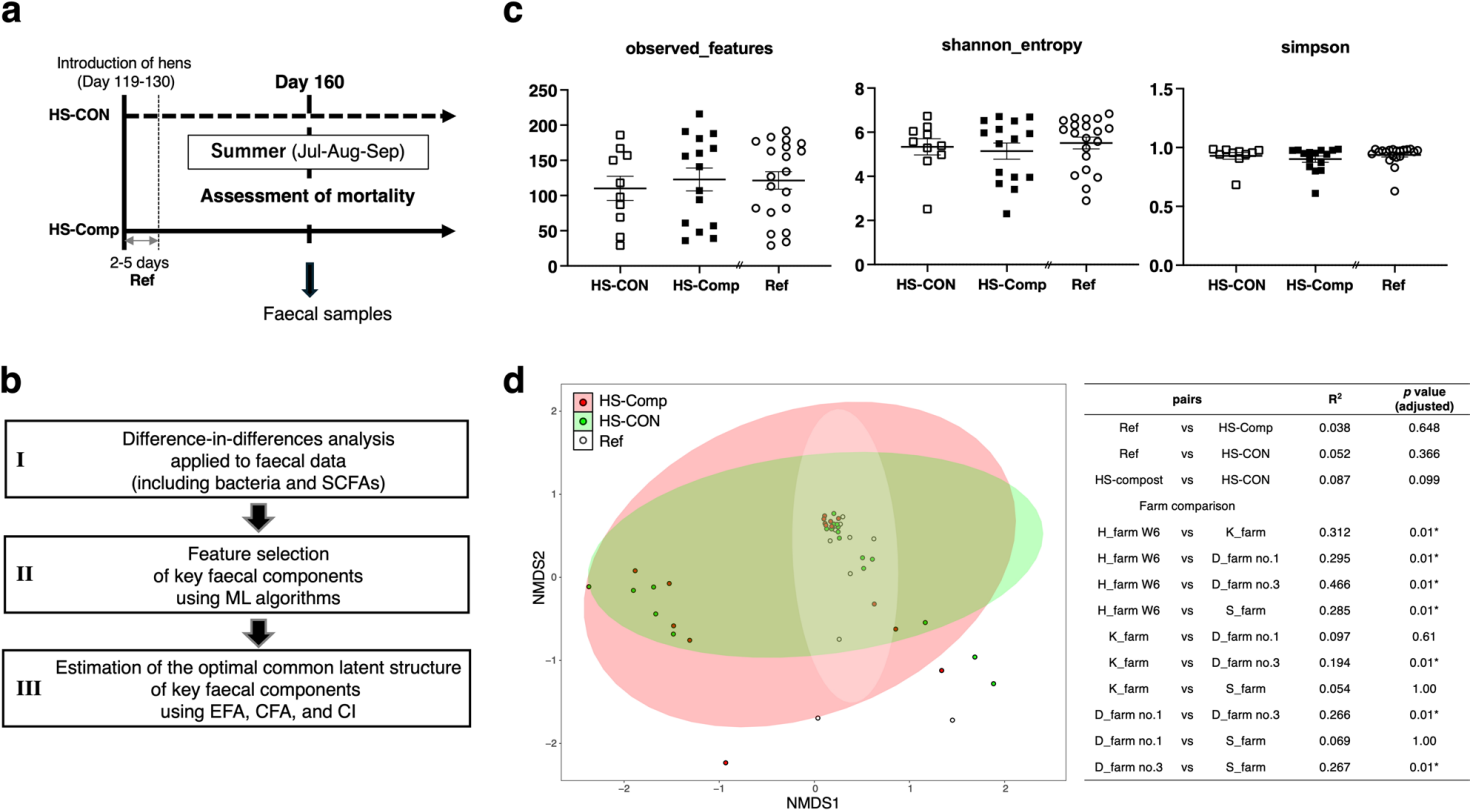
Relationships between the faecal bacterial diversity and mortality following compost administration. (**a**) Experimental schemes for different hen houses in 2016. The “Introduction” indicates the age of the laying hens at the time of introduction into the designated poultry house. The “2-5 days Ref” indicates the reference hen houses administered with the compost during the days 2–5 after the introduction. (**b**) Workflow for faecal bacterial evaluation under compost administration. (**c**) The number of observed features (OTUs), Shannon index, and Simpson index were used as α-diversity indices of the faecal bacterial populations. (**d**) Characteristics of the faecal bacteria of the cattle classified via nonmetric multidimensional scaling (NMDS). The NMDS data of the cattle were visualized for visual evaluation of β diversity. The data of “H_farm W6” (HS-Comp in 2016), “D_farm no.1” (HS-Comp in 2016), “D_farm no.3” (HS-Comp in 2016), “K_farm” (HS-CON in 2016), “Ref_D_farm no.1” (Reference in 2016), “Ref_D_farm no.3” (Reference in 2016) and “Ref_S_farm” (Reference in 2016) were compared

**Fig. 4:**
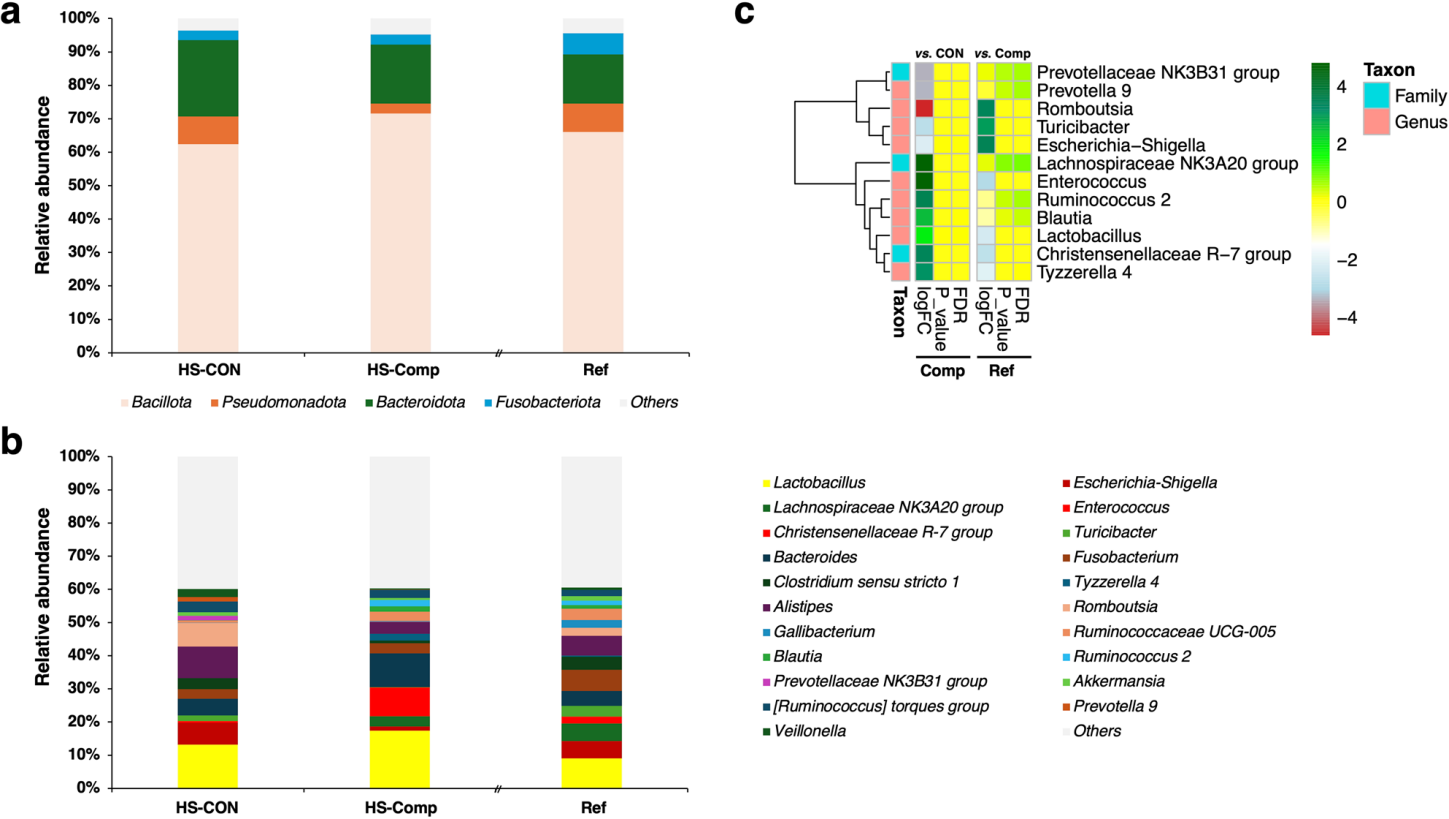
Faecal bacterial population on the tested farms. Relative abundance of (**a**) phyla and (**b**) genera (10% or more as the maximum relative abundance) in the faeces of the chickens. (**c**) The values of logFC, p value, and FDR (false discovery rate) of the faecal bacteria detected by differences-in-differences. The abbreviations used are as follows: CON, HS-CON group; Comp, HS-Comp group; Ref, reference in Fig. 3a

**Fig. 5:**
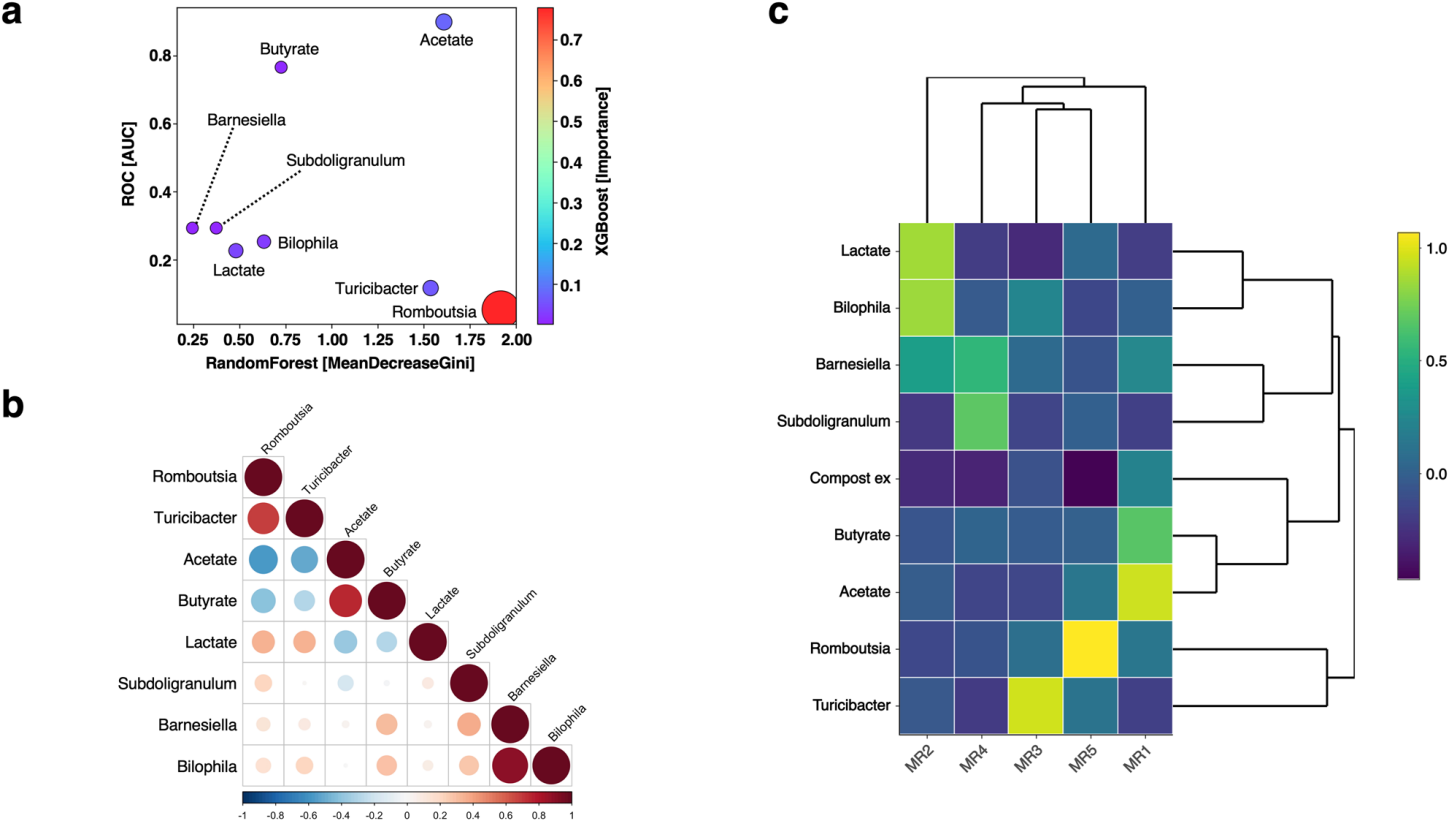
Evaluation of the feature component candidates using ML algorithms and EFA. (**a**) Relationship diagram of the feature components selected via three types of machine learning (ML) algorithms. The feature importance scores from the random forest, XGBoost, and ROC methods are visualized as a bubble chart. The abbreviations were as follows: AUC, area under the curve as a measure used in the ROC curve analysis; MeanDecreaseGini, a feature index of random forest; importance, a feature index of XGBoost. (**b**) Correlation heatmaps of the ML-selected components. Heatmaps based on data from the control group (HS-CON) and the compost administration group (HS-Comp) were generated. The correlation coefficients were determined by Spearman’s rank correlation function. (**c**) Heatmap of components obtained from exploratory factor analysis (EFA) with a factor number of 5. MR indicates the minimum residual method. The right bar shows the extents of the contribution values. The “Compost ex” indicates a condition in which the compost extract was administered

**Fig. 6:**
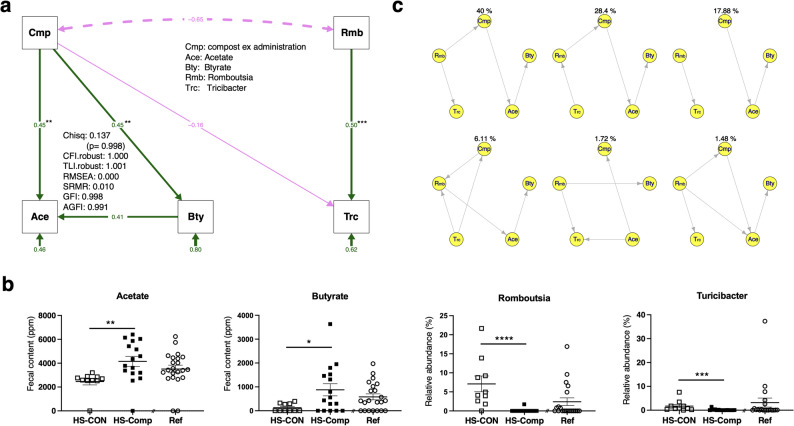
Estimation of the optimal component groups by structural equation. (**a**) The optimal model estimated by structural equation modelling (SEM) as a confirmatory factor analysis (CFA). The colours are as follows: green, putative positive interaction; purple, putative negative interaction. (**b**) The relative values of the SEM-selected components. *, *p* < 0.05; **, *p* < 0.01; and ***, *p* < 0.001. (**c**) Causal inference for the structural equation model computed by BayesLiNGAM. The DAGs (top six) based on BayesLiNGAM were visualized. The upper percentage of each DAG indicates the calculated ratio of the grouped DAGs. The abbreviations used are as follows: Cmp, treatment of the test group with the compost extract; Ace, acetate; Bty, butyrate; Rmb, the genus *Romboutsia*; and Trc, the genus *Turicibacter*. The main fit indices of the models are shown in the figures, and the other indices are shown. The asterisks indicate *p* values < 0.01. The abbreviations of the main fit indices are as follows: chisq, chi-square: χ^2^; *p* value, *p* value (chi-square); CFI. robust, robust comparative fix index; TLI. robust, robust Tucker–Lewis index; RMSEA, root mean square error of approximation; SRMR, standardized root mean residual; GFI, goodness-of-fit index; and AGFI, adjusted goodness-of-fit index

**Fig. 7:**
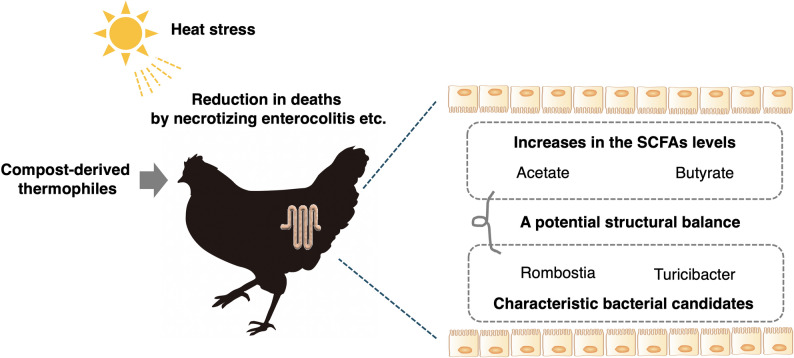
A putative model of the faecal environment associated with reduced mortality in hens under heat stress conditions

A further reproducibility survey was conducted on eight hen farms from July to September of the following year to elucidate the mechanism by which compost administration reduces mortality. In these observations, a total of 324,218 new egg-laying hens for HS-Comp group (approximately 119 ~ 130 days old) in 2016 were prepared and reared until 28 weeks after birth. Laying hens across six farms (the HS-Comp group in 2015 and five newly added hen farms) were administered compost, while hens on two farms served as controls and were not administered compost (HS-CON group in 2015) (Fig. 3a). The measured faecal bacterial composition and major gut metabolites (e.g., SCFAs) were analysed as shown in Fig. 3b using a combination of approaches, including difference-in-differences analysis, ML-based feature selection, exploratory and confirmatory factor analyses, and causal inference. The average temperatures in the HS-Comp group (max_temp, 31.36 ± 1.0 °C; min_temp, 23.53 ± 1.17 °C) were significantly greater (*p* = 0.027) than the average temperature in the HS-CON group (max_temp, 30.40 ± 1.96 °C; min_temp, 22.51 ± 1.14 °C) (Fig. S2). Under these conditions, the mortality rate in the HS-Comp group (0.083 ± 0.020%) tended to be lower than that in the HS-CON group (0.168 ± 0.163%) (*p* = 0.1563) (Fig. S3a). In particular, the mortality rates at 23–25 weeks of age were significantly lower in the HS-Comp group (0.083 ± 0.021%) than in the HS-CON group (0.295 ± 0.226%) (*p* = 0.0183). The OPG (oocysts per gram) values for each hen house did not necessarily decrease in August after the compost administration, although the values for farm D tended to be low (*p* = 0.1032 for farm no. 3; *p* = 0.0914 for farm D no. 1) (Fig. S3b). These results suggest that the reduced mortality among hens administered with compost extract was not necessarily associated with a reduction in OPG.

### Assessment of faecal bacterial composition and diversity

The α-diversity and β-diversity indices of the bacteria were examined (Fig. 3c and d). These results did not reveal a statistically significant difference between the control and compost groups. On the other hand, comparisons between farms revealed significant differences (Fig. 3d). Examination of the population of bacterial groups revealed no regularity at the phylum and genus levels at the farm level (Fig. S4) or in the availability of the compost (Fig. 4a and b). However, difference-in-differences (DID) analysis revealed common variations in the effects of compost administration (Fig. 4c). The levels of the genera *Enterococcus*, *Ruminococcus* 2, *Blautia*, *Lactobacillus*, and *Tyzzerella* 4 and the families *Lachnospiraceae* NK3A20 group and *Christensenellaceae* R-7 group were significantly increased by compost administration, but those of the genera *Prevotellaceae* NK3B31 group, *Prevotella* 9, *Romboutsia*, *Turicibacter*, and *Escherichia–Shigella* were significantly reduced (*p* < 0.05; FDR < 0.1). The levels of the genera *Enterococcus*, *Lactobacillus* and *Tyzzerella* 4 and the family *Christensenellaceae* R-7 group were significantly increased, whereas those of the genera *Escherichia–Shigella*, *Romboutsia*, and *Turicibacter* were significantly reduced by compost ex administration compared with those in the early days of compost administration (Ref) (*p* < 0.05; FDR < 0.1).

These results suggest that subtle changes in the bacterial population, which are difficult to observe in higher-level analyses, were observed after compost administration.

### Feature selection of faecal components

Next, feature selection via ML algorithms was performed to explore the common components for mortality reduction in the summer. First, twenty feature component candidates were selected via association analysis of the binarized whole dataset (Fig. S5a). The importance values of these feature component candidates were calculated via two types of ML algorithms: random forest (RF) (Fig. S5b) and eXtreme gradient boosting (XGBoost) (Fig. S5c). Feature components that could not necessarily be found by DID were selected and established a dataset with a non-Gaussian distribution (Table S1). In addition, the degree of the effect of compost administration was evaluated by the AUC by means of receiver operating characteristic (ROC) curve analysis (Table S1). As a result of the analysis, the genera *Barnesiella*, *Bilophila*, *Romboutsia*, *Subdoligranulum*, and *Turicibacter*, along with the metabolites acetate, butyrate, and lactate, were identified as key feature components via the ML algorithms (Fig. 5a). In particular, acetate and butyrate received high AUC ratings (0.900 and 0.767), respectively, according to the ROC curve analysis, in relation to compost administration (Table S1). Thus, these components were computationally estimated to be important factor candidates through feature selection using three types of ML algorithms and ROC curve analysis.

### Structural evaluation of faecal feature components using factor analyses

EFA was then performed to classify these components into computational clusters. First, the relationships between the components were evaluated using correlation analysis. Distinct clusters were identified (Fig. S6). In addition, since the values of correlation coefficients between these ML-selected components were not necessarily at a low level (|r|>0.4) (Fig. 5b), the function “promax”, an oblique rotation method, was adopted as the calculation condition to apply components with a high correlation coefficient. As shown in the scree plot (Fig. S7), “nfactor = 5” was applied as the number of very simple structures (VSS). An EFA heatmap was used to visualize the relationship between the administration of the compost extract and the ML-selected feature components (Fig. 5c). The values of the commonality indicator (h2) of these components were above 0.3 (Table S1). These results suggested a strong relationship between the administration of the compost extract and the faecal concentrations of the SCFAs acetate and butyrate.

Next, SEM was conducted as a confirmatory factor analysis (CFA) starting from the relationship with these factor groups. Because all the factors to be calculated were nonnormally distributed (Table S1) (Shapiro‒Wilk test, *p* < 0.05), the calculation conditions were established by robust estimation. An optimal model composed of the genera *Romboutsia* and *Turicibacter*, acetate and butyrate is shown in Fig. 6a. These statistical values were subsequently examined (Table S2). Among these components, acetate and butyrate were significantly increased by compost administration (*p* = 0.001 and *p* = 0.025, respectively), and the abundances of the genera *Romboutsia* and *Turicibacter* significantly decreased (*p* < 0.0001 and *p* = 0.001, respectively) (Fig. 6b). Additionally, the abundances of the genera *Barnesiella*, *Bilophila*, and *Subdoligranulum* and lactate in the ML-selected components tended to decrease with compost administration (*p* = 0.080; *p* = 0.034; *p* = 0.089; and *p* = 0.025, respectively) (Fig. S8). The values of the average causal mediation effect (ACME) and average direct effect (ADE), which are indices of the mediation relationships among the components of the optimal structural equation model obtained using causal mediation analysis (CMA), were not necessarily significant (Table S2). Specifically, the ACME of butyrate to acetate was significant, whereas that of Romboutsia to acetate was not significant. In other words, the importance of the group structure of the components in the optimal model was statistically evaluated using SEM and CMA. In causal inference analyses such as DirectLiNGAM (Fig. S9a) and BayesLiNGAM (Fig. 6c), the subset of components affecting SCFAs was visualized. On the basis of the results of the BayesLiNGAM analysis, it was also inferred that the effects of compost administration on acetate and butyrate were observed in the original network (87.76% within the top six relationships).

Thus, the relationships among a group of feature components characteristic of compost administration were computationally revealed. These relationships did not necessarily show a direct effect but rather a computational structural relationship. The results suggest that considering these components as functional groups was more informative than evaluating the changes in each component individually.

## Discussion

This study is the first to assess the induction of heat tolerance by oral administration of an extract of compost fermented with thermophiles. The administration of the compost extract reduced mortality under HS conditions in the summer to a level comparable to that of the non-HS group in autumn. According to the veterinarian’s instructions, laying hens that died were diagnosed with necrotic enteritis. Previous studies have reported that HS in poultry leads to increased intestinal permeability because of the disruption of tight junctions in both transcellular (intracellular) and paracellular (intercellular) pathways [16, 23], which may be associated with necrotic enteritis. Therefore, to evaluate the possible involvement of coccidia, which are known to be associated with necrotic enteritis, we assessed OPG values. However, the OPG values in the houses where the compost extract was administered were not necessarily reduced. To clarify the mechanism by which compost extract administration reduced mortality, we analysed the intestinal microbiota and its major metabolites, organic acids and inorganic acids. A trend of changes in microbial diversity was observed, with the composition of several microbial groups and the concentrations of faecal organic and inorganic acids changing as a result of compost application. In particular, decreases in the abundances of the bacterial genera *Romboutsia* and *Turicibacter*, along with increases in the concentrations of faecal acetate and butyrate, were identified as features of the optimal structural equation model. Considering the compositional nature of the microbiome and metabolite data, the results after dataset transformation (Table S2 and Figs. S9b, c and d) revealed largely consistent causal and mediation effects, with the main conclusions remaining unaffected.

Acetate and butyrate are generally known to have functions related to biological defence [24, 25]. Acetate enhances the intestinal defence mediated by epithelial cells, and it has been reported that acetate contributes to protecting mice against mortality induced by *E. coli* O157:H7 [26]. Moreover, acetate can influence the function of IgA [24]. In the present study, the abundance of the genus *Escherichia–Shigella* was reduced by the administration of the compost extract, which may be associated with increased faecal acetate levels. Butyrate increases the intestinal barrier by regulating the assembly of tight junctions [27]. Furthermore, butyrate promotes the differentiation of regulatory T (Treg) cells both in vitro and in vivo and alleviates colitis induced by adoptive transfer of CD4^+^CD45RB^hi^ T cells in *Rag1*^*−/−*^ mice [25]. The reduced mortality of heat-stressed hens administered compost extract was likely associated with increased production of acetate and butyrate in the intestine. A previous study reported an increase in butyrate levels at moderate temperatures in vitro under artificial anaerobic conditions associated with the administration of compost [28]. Our previous study [29] in beef calves revealed that oral administration of thermophilic probiotic bacteria increased the relative abundance of the phylum Bacteroidota and decreased that of Bacillota. Bacteroidota abundance was positively correlated with faecal propionate and butyrate concentrations, whereas this correlation was negative for Bacillota abundance. Furthermore, the same study demonstrated that the administration of thermophilic probiotics increased the abundance of the butyrate-producing family *Lachnospiraceae*. Similarly, in the present study, the bacterial groups whose abundance increased following the administration of the compost extract included butyrate-producing bacteria, such as those in the family *Lachnospiraceae* and the genus *Blautia*, as well as *Ruminococcus*, which degrades fibre and produces both acetate and butyrate. Therefore, in laying hens as well as in beef calves, the administration of thermophilic probiotics may increase intestinal acetate and butyrate production by driving an increase in the abundance of acetate- and butyrate-producing bacteria. Additionally, in the present study, the abundance of *Escherichia–Shigella* was reduced by compost administration, whereas that of the family *Christensenellaceae* R-7 was increased. A recent study reported that the *Christensenellaceae* R-7 group was positively correlated with total SCFA concentrations, whereas the *Escherichia–Shigella* group was negatively correlated with butyrate concentrations in broilers [30]. Therefore, alterations in the abundances of these bacterial groups may be associated with elevated concentrations of faecal acetate and butyrate.

In the present study, the relative abundances of the genera *Escherichia–Shigella* and *Turicibacter* decreased in hens administered compost. The genus *Escherichia–Shigella* species has been reported to be enriched in chickens with extremely severe necrotizing enterocolitis [31], consistent with the results of the present study. Similarly, the genus *Turicibacter* has been observed in growing broiler chickens under 50 days of age that developed necrotic enteritis [32]. Therefore, *Escherichia–Shigella* and *Turicibacter* are implicated in necrotic enteritis, and the decreased abundance of these bacterial groups is suggested to be associated with reduced mortality. The genus *Turicibacter* is widely distributed in the gut microbiota of various animals, including chickens [33, 34]. The effects of heat stress on *Turicibacter* vary among previous studies and across animal species. In the caecum digesta of pigs, the abundance of *Turicibacter* increased in response to heat stress [35]. In contrast, the abundance *Turicibacter* decreased under heat stress in hens [36] and dairy cows [37]. In a mouse model of dextran sodium sulfate (DSS)-induced colitis, the abundance of *Turicibacter* increased markedly as an inflammatory marker and decreased the level of faecal butyrate; however, pretreatment with glycerol monolaurate (GML) suppressed the increase in the abundance of *Turicibacter* and alleviated body weight loss and inflammatory responses [38]. Interestingly, in the same study, GML pretreatment led to increases in the abundances of the genera *Romboutsia*, *Lactobacillus*, and *Bifidobacterium*. In the present study, an increase in the abundance of the genus *Lactobacillus*, as well as a decrease in the abundance of the genus *Turicibacter*, was observed in hens administered the compost extract and may have contributed to the decreased mortality. Additionally, SEM revealed that a decrease in the abundance of *Turicibacter* caused by the administration of the compost extract was negatively associated with a decrease in the abundance of *Romboutsia* in the present study. In broilers that were administered *Weizmannia coagulans* (synonym: *Bacillus coagulans*), a thermostable probiotic lactic acid bacterium that is present in thermophile-fermented compost [39], the growth rate increased under HS conditions, and the abundance of the genus *Romboutsia* in faeces increased [40]. Therefore, changes in the genus *Romboutsia* may be involved in the probiotic-induced tolerance to HS. These findings suggest that the reduction in mortality following administration of the compost extract was associated with increased butyrate production and a decrease in the abundance of *Turicibacter*, which is related to changes in the abundance of *Romboutsia*. Notably, each farm housed a different commercial layer strain—Babcock B400 in the non-HS group, Julia lite in both the HS-Comp and HS-CON1 groups, and Julia in the HS-CON2 group. Therefore, it is possible that breed-specific characteristics contributed to the observed variation. In this study, the non-HS group served as a seasonal reference rather than a direct comparison group, whereas HS-CON2 provided an additional HS dataset from another strain to help contextualize the treatment effects across strains. Thus, although strain differences may have influenced the quantitative aspects of the responses, the overall pattern of HS mitigation associated with the compost extract remained consistent.

The PICRUSt2 algorithm was developed to predict functional pathways on the basis of bacterial 16 S RNA gene sequence data. Comparisons of the pathway data between the two treatments were conducted to assess the metabolic function of the faecal bacteriomes (Fig. S10). Volcano plot analysis revealed multiple significant changes in metabolic pathways associated with changes in the microbial community. Therefore, three pathways—PWY0–1338 (*p* < 0.001; FDR = 0.047), KDO–NAGLIPASYN–PWY (*p* < 0.001; FDR = 0.047), and AST–PWY (*p* < 0.001; FDR = 0.047)—were reduced by administration of the compost extract. PWY0–1338 is involved in the acquisition of polymyxin resistance in gram-negative bacteria such as *E. coli*, and its reduction may be related to the observed decrease in the abundance of *Escherichia–Shigella* in hens administered with the compost extract. AST–PWY is associated with the degradation of the carbon skeleton of L-arginine and is present in gram-negative bacteria such as *Pseudomonas* and *E. coli*. Additionally, KDO–NAGLIPASYN–PWY is involved in the biosynthetic pathway for lipid A, a component of lipopolysaccharides (LPS). Therefore, the reductions in AST–PWY and KDO–NAGLIPASYN–PWY appear to be associated with the decreased abundance of gram-negative bacteria, including *Escherichia–Shigella*.

This study demonstrated that the administration of an extract derived from thermophilically fermented compost to laying hens alleviated heat stress and reduced mortality (Fig. 7). The reduction in mortality associated with the compost extract was linked to alterations in the composition of the gut microbiota and the concentrations of SCFAs. In particular, compositional factor analysis using SEM produced a model in which the compost extract-induced increases in acetate and butyrate concentrations, along with decreases in the abundances of the genera *Romboutsia* and *Turicibacter*, best explained the observed outcomes. The idea of building a sustainable society is growing in importance, and strategies to promote survival in the face of global warming are also becoming increasingly critical. In this context, the ability to mitigate heat stress through the use of recycled products is particularly important. The alleviating effect of thermophilic probiotics on heat stress revealed in this study may be applicable to other livestock species; this topic warrants further investigation. In this study, 100-fold diluted compost extract was added to the drinking water at a concentration of ≤ 0.4% for continuous administration. Therefore, the energy and nutrient intake from the added compost extract is considered negligible. Our recent study revealed that the administration of thermophilic *Caldibacillus hisashii*, which is present in compost, altered the intestinal microbiota and improved feed efficiency in Japanese black calves [29]. At this time, the trend of increased abundance of members of phylum *Bacteroidota* (synonym: *Bacteroidetes*), which regulate SCFAs production, has been confirmed, as has the decreased abundance of methanogens. In other words, the administration of thermophilic probiotics itself may simultaneously control healthier intestinal metabolism and suppress the generation of greenhouse gases, potentially contributing to the heat tolerance of animals. Future research is expected to advance from these perspectives.

## Conclusion

In conclusion, oral administration of the thermophile-fermented compost extract alleviated heat stress induced-mortality in laying hens. These effects were associated with shifts in the composition of the gut microbiota, particularly decreases in *Romboutsia* and *Turicibacter* and increases in acetate and butyrate concentrations. Our findings highlight the potential of thermophilic probiotic bacteria to increase heat tolerance in poultry, offering a foundation for further research across diverse livestock species.

## Methods

### Hen management

Hen-rearing trials were conducted at dedicated farms of Crest Co., Ltd., which are located in Aichi (N34.91, E136.88), Gifu (N35.44, E136.98 and N35.53, E136.98), Hyogo (N34.88, E134.78), and Okayama (N35.08, E133.89). Eleven hen houses were used in this study. Three hen strains (Julia, Julia lite, or Babcock B400) were conventionally maintained at one experimental hen house. All chickens were orally administered commercial feeds and tap water *ad libitum* in accordance with the farm-specific guidelines. The commercial feed provided was identical across all the farms, and its ingredients consisted of 51% grains, 27% vegetable oil meal, 5% milling byproducts, 1% animal-derived feedstuffs, and 16% other components. All hens were managed under a photoperiod consisting of 16 h of light (04:00 to 20:00) and 8 h of darkness. Eleven groups of adult chickens, each comprising different strains that were not necessarily the same across different hen houses, were maintained with in-house blended feed available from approximately day 120 after birth on the following farms: H_farm W2 (non-HS group in 2015), strain Babcock B400, *n* = 54,488/hen house at the initial date; H_farm W1 (HS-Comp in 2015), strain Julia lite, *n* = 55,537/hen house at the initial date; M_farm W3 (HS-CON1 in 2015), strain Julia lite, *n* = 55,618/hen house at the initial date; M_farm W5 (HS-CON2 in 2015), strain Julia lite, *n* = 55,613/hen house at the initial date; H_farm W6 (HS-Comp in 2016), strain Babcock B400, *n* = 64,239/hen house at the initial date; K_farm A (HS-CON in 2016), strain Julia lite, *n* = 28,000/hen house at the initial date; K_farm B (HS-CON in 2016), strain Julia lite, *n* = 28,000/hen house at the initial date; D_farm no. 1 (HS-Comp in 2016), strain Julia lite, *n* = 74,389/hen house at the initial date; D_farm no. 3 (HS-Comp in 2016), strain Julia lite, *n* = 65,433/hen house at the initial date; O_farm (HS-Comp in 2016), strain Babcock B400, *n* = 55,374/hen house at the initial date; and S_farm no. 8 (HS-Comp in 2016), strain Julia lite, *n* = 64,783/hen house at the initial date. All animal experiments were carried out in accordance with the guidelines of the farms listed below and the Institutional Animal Care and Use Committee of Chiba University (approval numbers: DOU27-131 and DOU28-157).

### Preparation of compost-derived *Bacillaceae* as a feed additive

Fermented compost from marine animal resources (hereafter referred to as compost) was prepared via an aerobic repeated fed-batch fermentation system at high temperatures (approximately 75 °C) (fermentation-associated self-heating), as previously described [41] (Miroku Co. Ltd., and Keiyo Gas Energy Solution Co. Ltd., Japan). The powdered compost and/or its extract were used as appropriate for the feeding conditions of the experimental animals. A compost extract solution was prepared by diluting the compost 1/100 with potable water (vol./vol.) and incubated under aerobic conditions at 60 °C for at least 10 h (this solution was used as the compost extract) [17–22].

The compost extract was adjusted to contain 10^2^–10^3^ CFU/mL *C. hisashii* N-11 strain [42] and *Weizmannia coagulans* (synonyms: *Heyndrickxia coagulance* and *Bacillus coagulans*) N-16 strains [20] isolated via a germ-free/gnotobiot experiment [39]. As previously reported [17–22], the *C. hisashii* N-11 suspension was spread onto heart infusion agar (Shimadzu Co., Ltd., Kyoto, Japan) and cultured at 55 °C. The suspension of *Weizmannia coagulan* N-16 was spread onto tryptic soy broth (TSB) agar (Merk Life Science G.K., Tokyo, Japan) and cultured at 40 °C. The cultivated bacteria were added to the diluted compost extract (1/100 of the compost powder) (Miroku Co., Ltd., Oita, Japan, and Keiyo Gas Energy Solution Co., Ltd., Chiba, Japan). A compost extract comprising a 1:1 mixture of the two strains, adjusted to 10^2^–10^3^ CFU/mL in portable water, was orally administered *ad libitum*. In brief, the compost extract was administered in 0.5% drinking water, resulting in adjusted amounts of probiotic strains.

*C. hisashii* and *W. coagulans* N-16 strains, which are compost-derived *Bacillaceae* species in the compost solution, were prepared by Sermas Co., Ltd. (Chiba, Japan). *C. hisashii*, which was initially isolated as the *Bacillus thermoamylovorans* N11 strain [20], was registered as *B. hisashii* (type strain N-11^T^ = NRBC 110226^T^ = LMG 28201^T^) [39] and under accession number NITE BP-863 given by the international depositary authority at the National Institute of Technology and Evolution (NITE) in Japan (Sep 26, 2014); it was recently reclassified as *C. hisashii* [43] and cultured as previously described [20]. Similarly, the *W. coagulans* N-16 strain was registered under accession number NITE BP-02066.

### Preparation of faecal samples

Faeces were collected by random sampling from the hen houses described in the “Hen management” section of the Materials and Methods. Specifically, each test sample was prepared in the following groups: HS-CON in 2016 (K_farm A and K_farm B) as the control group, which were not administered the compost extract; HS-Comp in 2016 (H_farm W6, D_farm no. 1, and D_farm no. 3), which were administered the compost extract for 30 days; and Ref (D_farm no. 1, D_farm no. 3, and S_farm no. 8), which were administered the compost extract for 5 days. The ages of the hens in each group are listed in the figure legend (Fig. S1). All the faecal samples obtained for the analyses were transported at -20 °C and subsequently stored at -60 °C to -80 °C until the subsequent omics analyses were performed. Sequencing of the bacterial 16 S RNA gene and metabolomic analyses were performed via the procedures described below.

### 16 S rRNA sequencing and analysis of faecal samples

Total DNA was obtained from faeces in accordance with previously described methods [29]. 16 S rRNA gene analysis was carried out via PCR amplification with primers targeting the V4 region (515 F–806R) of the 16 S rRNA gene according to a previous report [29]. The PCR mixture contained 4 µL of DNA template, 0.5 µL of ExTaq polymerase (Takara Bio Inc., Kusatsu, Japan), 5 µL of ExTaq buffer, 5 µL of dNTPs, and 10 pmol each of the forward and reverse primers and was brought to a total volume of 50 µL with DNA-free water. Amplicons were generated with 30 cycles of 98 °C for 30 s, 55 °C for 45 s, and 72 °C for 2 min and were subsequently purified using AMPure XP (Beckman Coulter Inc., Brea, CA, USA). Multiplexed amplicon sequences for the samples were sequenced via the MiSeq platform according to the manufacturer’s instructions. The obtained FASTAQ format files were analysed using QIIME2 (https://qiime2.org) [44]. Additionally, the sequences were trimmed via maxEE (default: inf) of the DADA2 plugin, and the trimmed sequences were taxonomically classified via vsearch (using the SILVA database). Functional pathway predictions were performed using the PICRUSt2 algorithm (https://github.com/picrust/picrust2), which estimates the potential metabolic capabilities of the microbial communities based on 16S rRNA gene sequences. All 16S rRNA sequence datasets were deposited in the GenBank Sequence Read Archive database, as described in the “Data and materials availability” section. Statistical analysis of nonmetric multidimensional scaling (NMDS) values was performed using the “vegan” (distance = bray as the calculation condition) and “pairwiseAdonis” libraries in R software. The plot was generated using the “ggplot2” library.

### Metabolite analysis

The concentrations of SCFAs (acetate, propionate, and butyrate), lactate, and succinate in the frozen fresh faecal samples were determined using an HPLC Prominence instrument equipped with an electrical conductivity detector (CDD-10A_VP_) (Shimazu, Japan) [28] according to the manufacturer’s protocol, with some modifications. In brief, the faecal samples (200–400 mg) were mixed with a 9-fold volume of Milli-Q water for 10 min. After centrifugation at 15,000 rpm, the supernatant was filtered through a 0.45-µm Millex-HA filter (SLHA033SS) (Millipore, USA). The filtered solutions were prepared for HPLC analysis. HPLC analysis was carried out on an ion-exclusion column (Shim-pack SCR-102 H) (Shimazu, Japan). The measurement conditions were as follows: mobile phase, 5 mM *p*-toluenesulfonic acid; buffer, 5 mM *p*-toluenesulfonic acid, 20 mM Bis-Tris, and 0.2 mM EDTA-4 H; temperature setting of the instrument, 40 °C; and flow rate, 0.8 mL/min. Data processing was performed using LabSolutions Insight software (Shimadzu).

### Feature selection

Association analysis (AA), an elementary unsupervised ML method, was applied [45, 46]. In brief, the faecal omics data were classified on the basis of the median value (M) of the data themselves and sorted as 0 (< M) or 1 (> M). The library packages “arules” and “arulesViz” of R software were used to conduct association rule mining in these datasets. Furthermore, to extract feature factors via supervised ML, random forest [47], a type of ML with bagging (bootstrap aggregating) [48], was applied using the R software package “randomForest” (https://cran.r-project.org/web/packages/randomForest/index.html). XGBoost (eXtreme Gradient Boosting) [49], a type of ML algorithm with extreme gradient boosting [48], was applied using the R software package “xgboost” (https://cran.r-project.org/web/packages/xgboost/index.html). These algorithms were used to select the feature components from the whole dataset as previously reported [50–53]. To evaluate the diagnostic accuracy of the feature components, receiver operating characteristic (ROC) curve analysis was performed using the R library “Epi”. The conditions for administering the compost extract were set to 1. The calculated area under the curve (AUC) was used as the classification indicator. The feature importance scores from random forest, XGBoost, and ROC were visualized as a bubble chart using the python software packages “matplotlib”, “pandas”, and “numpy” as described on the website (https://matplotlib.org/stable/index.html).

### Exploratory factor analysis

In brief, we conducted a search for important factors via exploratory factor analysis (EFA) [54, 55] conducted with the library packages “psych” and “GPArotation” in R software. The analysis codes were obtained from the website (https://cran.r-project.org/web/packages/psych/index.html). The number of factors and their values were calculated by the function ‘VSS’ for a very simple structure (VSS). In this study, EFA was performed with “fm = minres” as the condition, that is, on the basis of the minimum residual (MR) method. Additionally, correlation analysis was performed by the library packages “corrplot”, “RcolorBrewer”, and “Hmisc” in R software. In this study, Spearman’s rank correlation coefficients were calculated to determine whether an oblique rotation or an orthogonal rotation should be applied as the calculation method. The other values for EFA were as follows: h2, communality score; u2, uniqueness score; and com, complexity, an information score that is generally related to uniqueness. The selected factors calculated by the function ‘fa’ were visualized via the library package “heatmaply” of R software (https://cran.r-project.org/web/packages/heatmaply/vignettes/heatmaply.html).

### Data preprocessing

For the structural equation modelling (SEM), mediation analysis (CMA), DirectLiNGAM, and BayesLiNGAM, the datasets were preprocessed to account for their characteristics. To account for their compositional nature, microbiome relative abundance data were transformed using the centred log-ratio (CLR) method, whereas metabolic data were transformed using a logarithmic scale as described in previous reports [56, 57].

### Structural equation modelling

To assess the structural similarity across the different hen houses, SEM for confirmatory factor analysis (CFA) was performed for a group of components with many directed acyclic graphs (DAGs) in accordance with previous reports [58–60]. For the selected components, SEM was conducted using the package “lavaan” [61] in R software. Analysis codes are based on those on the website (https://lavaan.ugent.be). As CFA requires a hypothesis, the components selected by EFA after ML were utilized as factors for a latent construct of metabolites and the microbiota. The distribution of the data was assessed using the Shapiro‒Wilk test in the R library “MVN” to determine whether it was Gaussian or non-Gaussian. In this study, the hypothesized models were statistically estimated via the robust maximum likelihood parameter estimator ‘MLR’ (for non-Gaussian data distribution) as a computational condition for the R libraries “lavaan” and “sem”. Model fit was assessed by the chi-square *p* value (*p* > 0.05, nonsignificant), robust comparative fit index (CFI.rbust/cfi.rbust) (> 0.95), robust Tucker–Lewis index (TLI.rbust/tli.rbust) (> 0.95), root mean square error of approximation (RMSEA/rmsea) (< 0.07), standardized root mean residual (SRMR/srmr) (< 0.08), goodness-of-fit index (GFI/gfi) (> 0.95), and adjusted goodness-of-fit index (AGFI/agfi) (> 0.95) as indices of good model fit [62]. Additionally, standardized residuals (SRs) (|SR|<2.58) were complementarily confirmed. To select the optimal structural equation, when the above conditions were met, an equation with little difference between the GFI and AGFI values was adopted. Next, the model with a low value of the Akaike information criterion (AIC) was adopted from among many candidate models. Standard errors were calculated via 1000 bootstrap draws via the maximum likelihood method. Path diagrams of the good model were constructed with layout="tree” using the “semPlot” package of R software [63].

### Causal inference

To assess the importance as a group of feature components, causal mediation was analysed using “mediation” [64], a package in R software based on a tutorial website (https://rpubs.com/Momen/485122*).* After each regression relationship with “~” in the selected model was assessed using the “lm” function, the values of the mediation relationships between the components of the optimal structural equation model were evaluated for the estimated average causal mediation effect (ACME) and average direct effect (ADE), and the proportion of the total effect through mediation was calculated by nonparametric bootstrapping (“boot = TRUE”) with “sims = 1000” as the number of iterative calculations. A direct method for learning a linear non-Gaussian acyclic structural equation model (DirectLiNGAM) was applied to infer structural models considering the unobserved data beyond the limited distribution of experimental data (https://lingam.readthedocs.io/en/latest/reference/bottom_up_parce_lingam.html). DirectLiNGAM was built in Python (version 3.10.8) via Mac OS Sequoia (version 15.3). The data calculated by the DirectLiNGAM analysis were analysed using the Python libraries numpy (version 1.26.4), pandas (version 2.2.3), matplotlib (version 3.9.2), cikit-learn (version 1.6.1), lingam (version 1.8.0), and “graphviz (version 0.19.1)” to ensure the validity of the acyclic graphs (DAGs). The BayesLiNGAM method, a Bayesian score-based approach, was applied for causal structural inference among the components in the optimal model as previously described [59, 60]. On the basis of information on the website (https://www.cs.helsinki.fi/group/neuroinf/lingam/bayeslingam/), the BayesLiNGAM was established by the “fastICA” package (https://cran.r-project.org/web/packages/fastICA) of R software. The percentage data calculated by BayesLiNGAM were visualized with the R library package “igraph” as previously described [59, 60].

### Statistical analyses

Data for frequentist equivalence testing were analysed as follows: the Jarque–Bera test or Shapiro‒Wilk test was used to evaluate the Gaussian or non-Gaussian nature of the distributions and select parametric and nonparametric analyses. The Jarque–Bera test was performed on the “tseries” library of R software. The Shapiro‒Wilk test was performed using the R software function “shapiro.test” and the R library “MVN”. The F test was used to evaluate the homogeneity of variance and to determine whether the unpaired t test or Welch’s t test was conducted for parametric analysis. The Wilcoxon rank-sum test and Wilcoxon signed-rank test were conducted for nonparametric analysis. Additionally, ANOVA followed by Tukey’s HSD test and the Kruskal‒Wallis test followed by the Steel‒Dwass test and/or Dunn test, as appropriate methods dependent upon datasets, were performed. The Steel–Dwass test and Dunn test were performed using the “NSM3” library and “dunn.test” library of R software, respectively. The threshold for statistical significance was *p* < 0.05, and marginal significance was assumed at 0.05 ≤ *p* < 0.20. The data were analysed via R software (version 4.3.3 and 4.5.1). Prism software (version 10) and Microsoft Office (version 16) were also used. The data are presented as the means ± SEs. Data collection and analyses were not performed in a blinded manner.

## Supplementary Information

Below is the link to the electronic supplementary material.

Supplementary Material 1

## Data Availability

Raw files of the bacterial V4 16 S rRNA sequences were deposited in the DNA Data Bank of Japan (DDBJ) under NCBI BioProject accession number DRA021308 (experiment: DRX665980-DRX666024). All the data were stored in a source data file (“Data_hen_final_new.xlsx”). Optional command files and the related information were stored on the figshare site and registered (10.6084/m9.figshare.30183436). Please contact the corresponding authors to obtain any additional information
