# Supplementary material for: Compost fermented with thermophilic Bacillaceae reduces heat stress–induced mortality in laying hens through gut microbial modulation
Source: Anim Microbiome. 2026 Feb 3;8:9. doi: 10.1186/s42523-026-00520-5 (PMC12870368; doi:10.1186/s42523-026-00520-5)
Supplement: Supplementary file 1 — Supplementary Material 1 [file 42523_2026_520_MOESM1_ESM.pdf]

Supplementary Information

Compost fermented with thermophilic Bacillaceae reduces heat stress–induced mortality in laying hens through gut microbial modulation

Yudai Inabu[1,2], Hirokuni Miyamoto[3,4,5,6,7*], Hideyuki Takahashi[1*], Tamotsu Kato[5], Shigeharu Moriya[8], Atsushi Kurotani[9], Teruno Nakaguma[2,6,7], Naoko Tsuji[7], Chie Ishii[5,7], Makiko Matsuura[3,7], Satoshi Wada[9], Takashi Satoh[10], Motoaki Udagawa[11], Hisashi Miyamoto[12], Jun Kikuchi[12], Hiroaki Kodama[3], Hiroshi Ohno[5*]

[1]Kuju Agricultural Research Center, Graduate School of Agriculture, Kyushu University, Oita, Japan, 878-0201
[2]Graduate School of Integrated Sciences for Life, Hiroshima University, Hiroshima, Japan
[3]Graduate School of Horticulture, Chiba University, Matsudo, Japan, 271-8501
[4]Graduate School of Medical Life Science, Yokohama City University, Tsurumi, Yokohama 230-0045, Japan
[5]RIKEN Center for Integrative Medical Sciences, Yokohama, Kanagawa, Japan, 230-0045
[6]Japan Eco-science (Nikkan Kagaku) Co., Ltd., Chiba, Japan, 260-0034
[7]Sermas, Co., Ltd., Chiba, Japan, 271-8501
[8]RIKEN, Center for Advanced Photonics, Wako, Saitama, 351-0198, Japan
[9]Research Center for Agricultural Information Technology, National Agriculture and Food Research Organization, Tsukuba, Ibaraki, Japan, 305-0856
[10] Division of Hematology, Kitasato University School of Allied Health Sciences, Sagamihara, Kanagawa 252-0373, Japan
[11] Keiyo Gas Energy Solution Co., Ltd., Ichikawa, Chiba 272-0033, Japan
[12] Miroku Co., Ltd., Kitsuki, Oita 873-0021, Japan
[13]RIKEN Center for Sustainable Resource Science, Yokohama, Kanagawa, Japan, 230-0045

*Corresponding author:
Hirokuni Miyamoto, RIKEN Integrated Medical Science Center, Yokohama, Kanagawa, Japan, 230-0045.
Email: hirokuni.miyamoto@riken.jp
Hideyuki Takahashi, Kuju Agricultural Research Center, Graduate School of Agriculture, Kyushu University, Taketa, Oita 878-0201, Japan.
Email: takahashi.hideyuki.990@m.kyushu-u.ac.jp
Hiroshi Ohno, RIKEN Integrated Medical Science Center, Yokohama, Kanagawa, Japan, 230-0045.
Email: hirokuni.miyamoto@riken.jp

Contents

Fig.S1 Relationships between growth stage and mortality in chickens.

Fig.S2 Relationships between the maximum and minimum temperatures of farms for reproducibility testing

Fig.S3 Death rates in farms for reproducibility testing and OPG values in the faeces

Fig.S4 Faecal bacterial populations at each farm

Fig.S5 Feature importance rankings of faecal components selected via ML algorithms.

Fig.S6 Clustering heatmaps of the correlations between ML-selected faecal components.

Fig.S7 Evaluation by exploratory factor analysis (EFA) for the feature components.

Fig.S8 Relative values of the other EFA components excluding SEM-selected components.

Fig.S9 Validation of the optimal structural equation model by DirectLiNGAM.

Fig.S10 Volcano plot for pathways identified at 160 days of age.

Table S1 Statistical values for EFA.

Table S2 Statistical values of the final optimal structural equation models

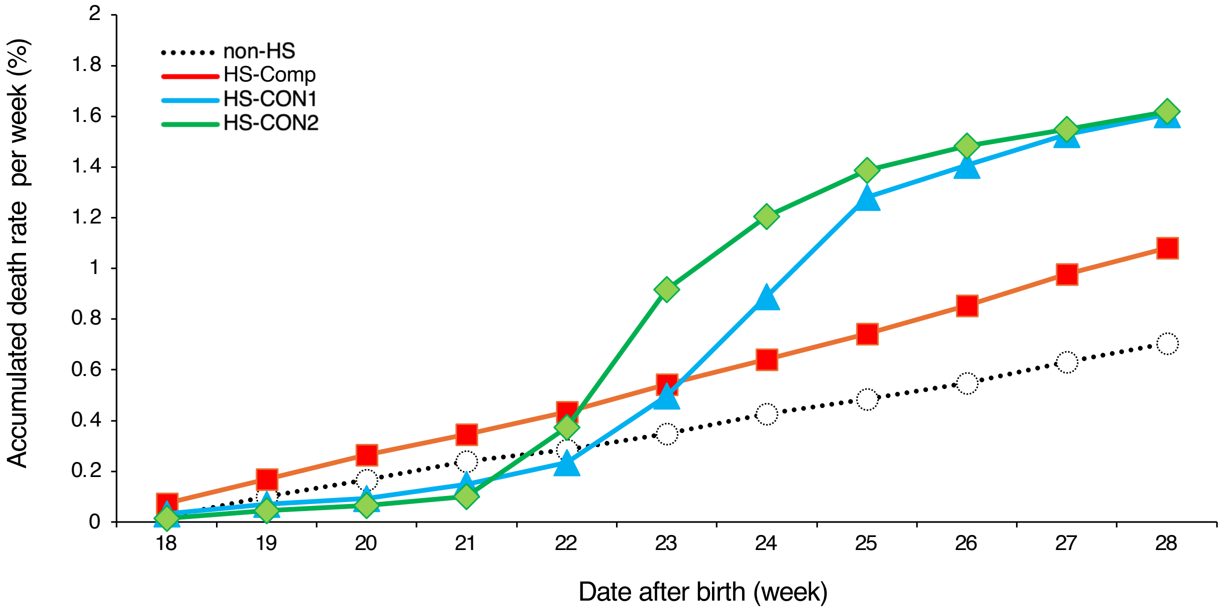

Fig.S1

Relationships between growth stage and mortality in chickens.

The mortality rates shown in Fig. 2b are the cumulative data per week. The “non-HS” indicates the data from the rearing period in early autumn. Three groups were established according to the compost application conditions in July and August of 2015. The “HS-Comp” indicates the group administered with the compost extract; the “HS-CON1” and “HS-CON2” are groups not administered with the compost. Significance is indicated as follows: #, $p<0.1$; *, $p<0.05$; **, $p<0.01$; and ***, $p<0.001$.

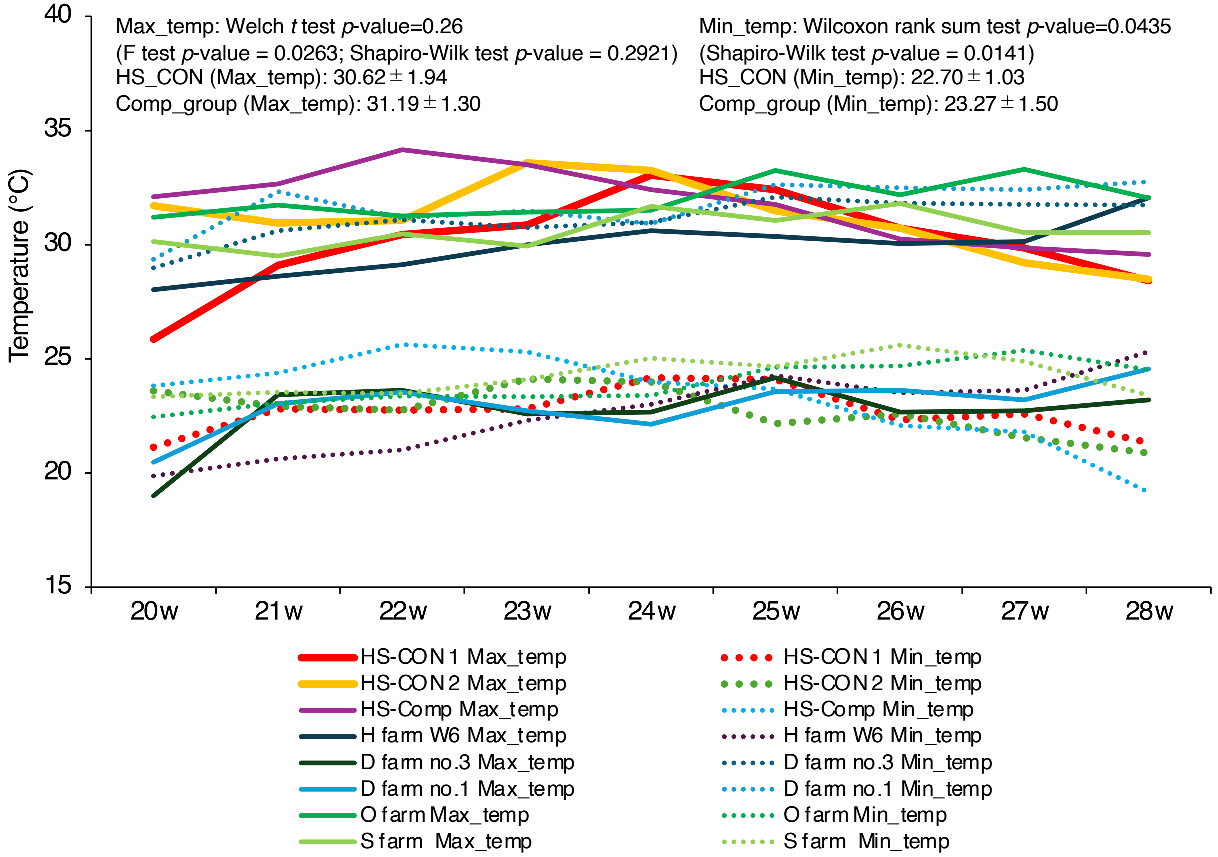

Fig.S2

Relationships between the maximum and minimum temperatures in farms for reproducibility testing

The relationships between the temperatures of the previous year and the next year are shown. The "H_farm", "K_farm", "D_farm", "O_farm", and "S_farm" represent each farm in the compost ex group. The data from "HS-CON1" (M farm W3) and "HS-CON2" (M farm W5), as well as "HS-Comp" (H farm W1) in 2015 are shown for comparison, and the data from 2016 for the other farms (HS-comp treated group in 2016) are shown.

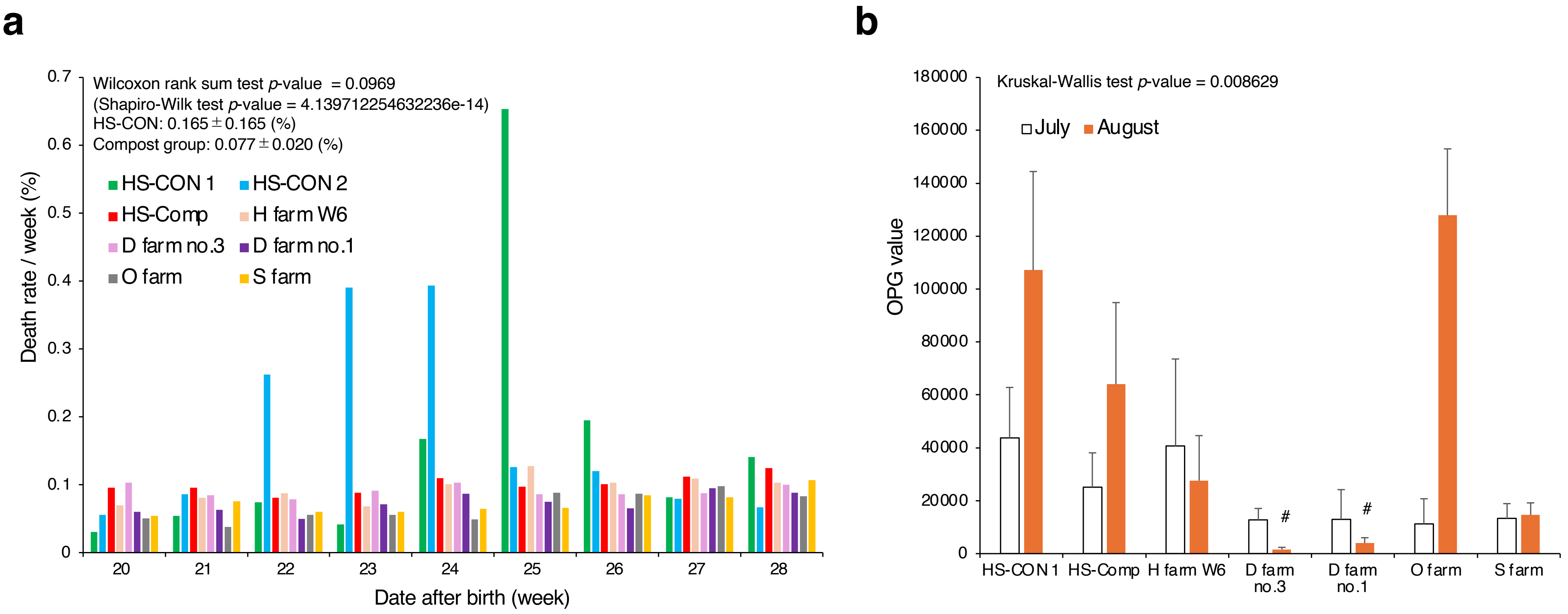

Fig.S3

Death rates in farms for reproducibility testing and OPG values in the faeces

(a) Death rates in the different hen farms in Fig. S2. The "H_farm", "D_farm", "O_farm", and "S_farm" represent each farm in the compost ex group. (b) The values of OPG (Oocysts per gram) for each hen house in July and August are shown. The data from "HS-CON1" (M farm W3) and "HS-CON2" (M farm W5), as well as "HS-Comp" (H farm W1) in 2015 are shown as comparison, and the data from 2016 for the other farms (HS-comp treated group in 2016) are shown. Significance is indicated as follows: #, $p<0.2$.

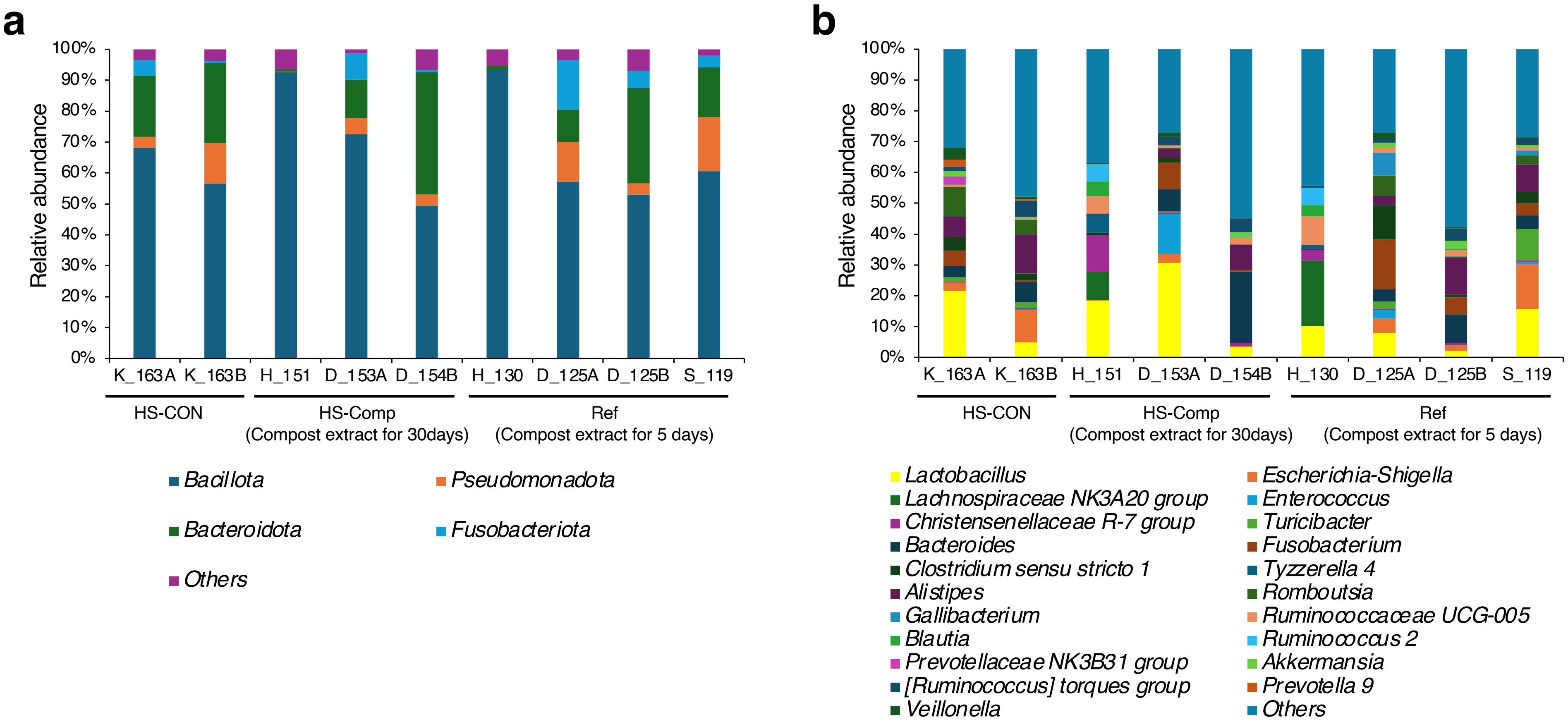

Fig.S4

Faecal bacterial populations at each farm

Relative abundance of faecal bacteria (a) at the phylum level and (b) at the genus level at each farm. The "HS-CON" (HS-CON in 2016), "HS-Comp (compost extract for 30days)"(HS-Comp treated group in 2016), and "Ref" (compost extract for 5 days)" (reference group in 2016) indicate HS-CON, HS-Comp, and Ref in Fig. 3a, respectively. The format of the names on the X axis is [farm name]_[age] and the hen house mark as follows: K_163A, 163-day-old hens on K_farm, hen house mark A; K_163B, 163-day-old hens on K_farm, hen house mark B; H_151, 151-day-old hens on H_farm; D_153A, 153-day-old hens on D_farm no.1, hen house mark A; D_154B, 154-day-old hens on D_farm no.3, hen house mark B; H_130, 130-day-old hens on H_farm; D_125A, 125-day-old hens on D_farm no.1, hen house mark A; D_125B, 125-day-old hens on D_farm no.3, hen house mark B; S_119, 119-day-old hens on S_farm.

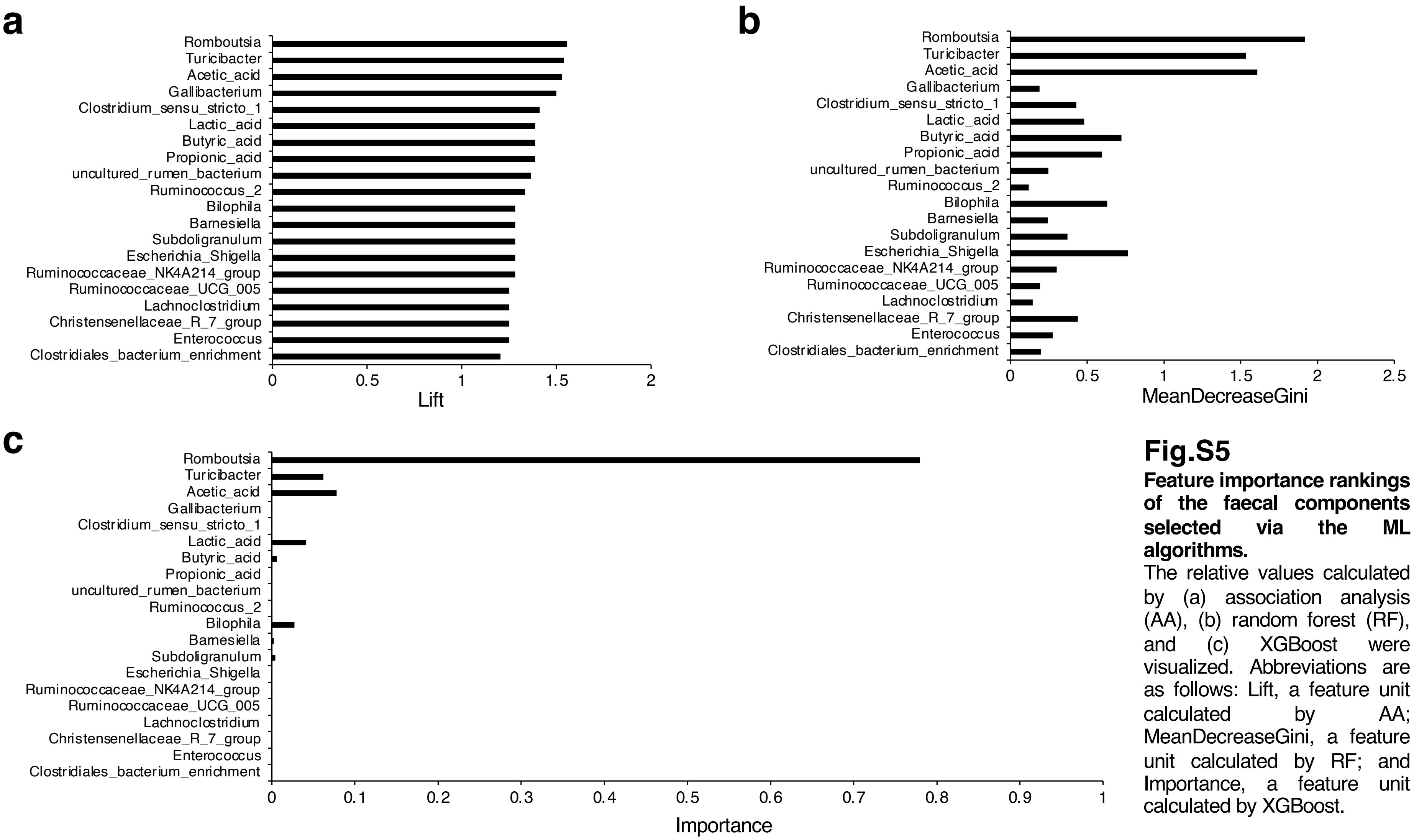

Fig.S5

Feature importance rankings of the faecal components selected via the ML algorithms.
The relative values calculated by (a) association analysis (AA), (b) random forest (RF), and (c) XGBoost were visualized. Abbreviations are as follows: Lift, a feature unit calculated by AA; MeanDecreaseGini, a feature unit calculated by RF; and Importance, a feature unit calculated by XGBoost.

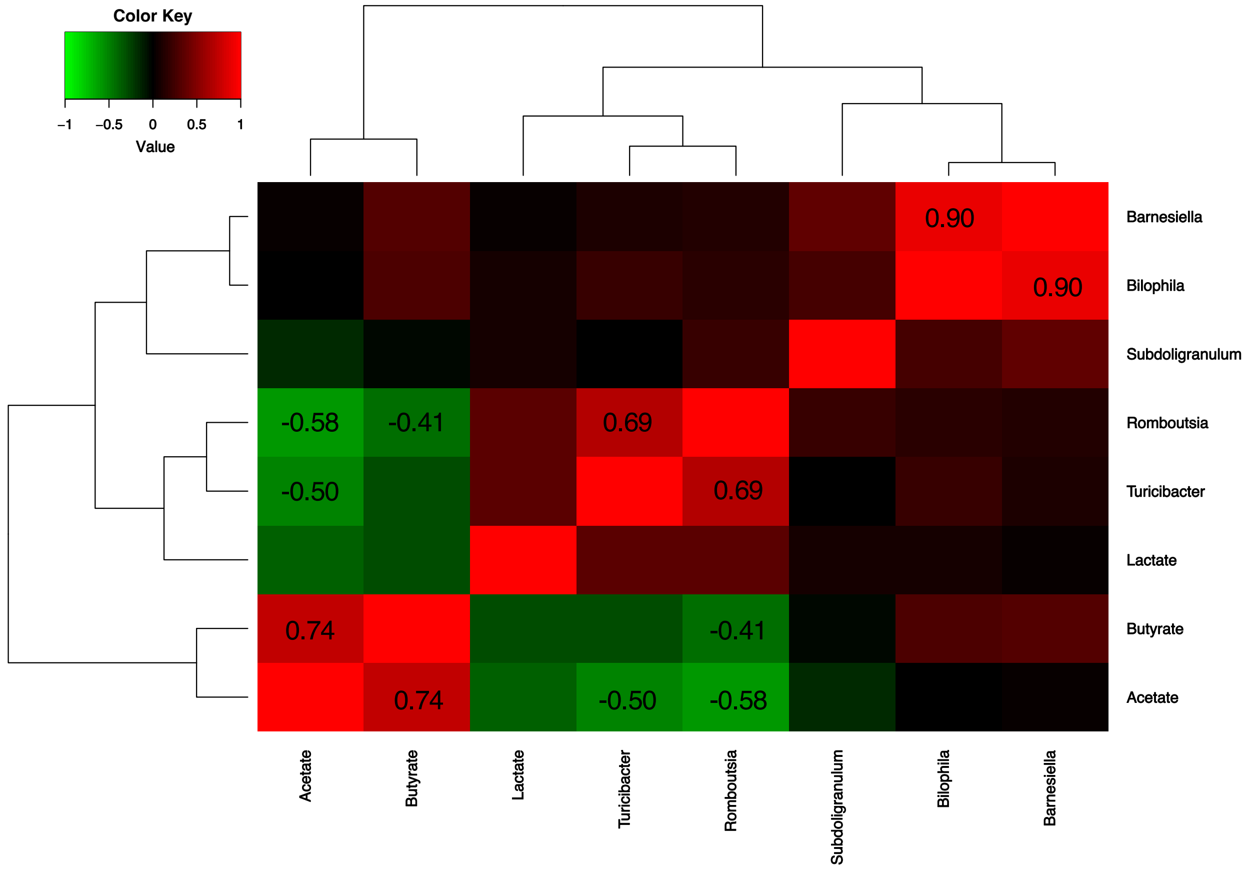

Fig.S6

Clustering heatmaps of the correlations between ML-selected faecal components.

Heatmaps based on data from the control group (HS-CON) and the compost administration (HS-Comp) group were generated. The numbers in the columns represent the values of the correlation coefficients (|r|>0.4) between the overlapping components.

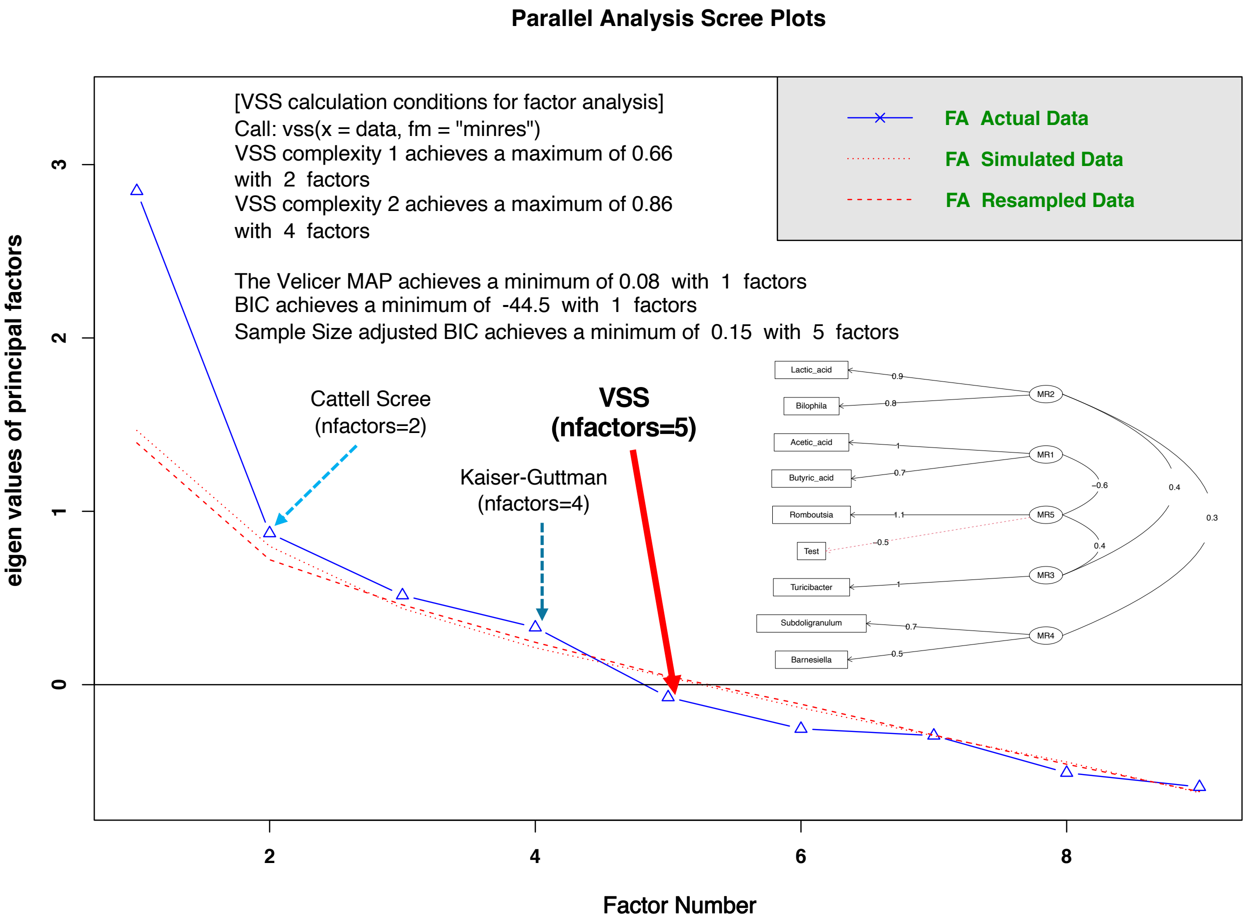

Fig. S7

Evaluation by exploratory factor analysis (EFA) for the feature components.

The panel shows the parallel analysis scree plots for EFA. The arrow designed ‘Cattle Scree’ (blue broken line) indicates the number of factors based on the Cattell scree criteria. The arrow point marked with ‘Kaiser–Guttman’ (brown broken line) indicates the number of factors based on the Kaiser–Guttman criterion. The arrow point marked with ‘VSS’ (red line) indicates the number of factors on the basis of the VSS-calculated criteria. The directed graph within the scree plot shows a diagram of factor analysis (nfactor=5). Abbreviations are as follows: FA, factor analysis; VSS, very simple structure; BIC, Bayesian information criterion; MR, minres (minimum residual) method; fa, calculation function for EFA.

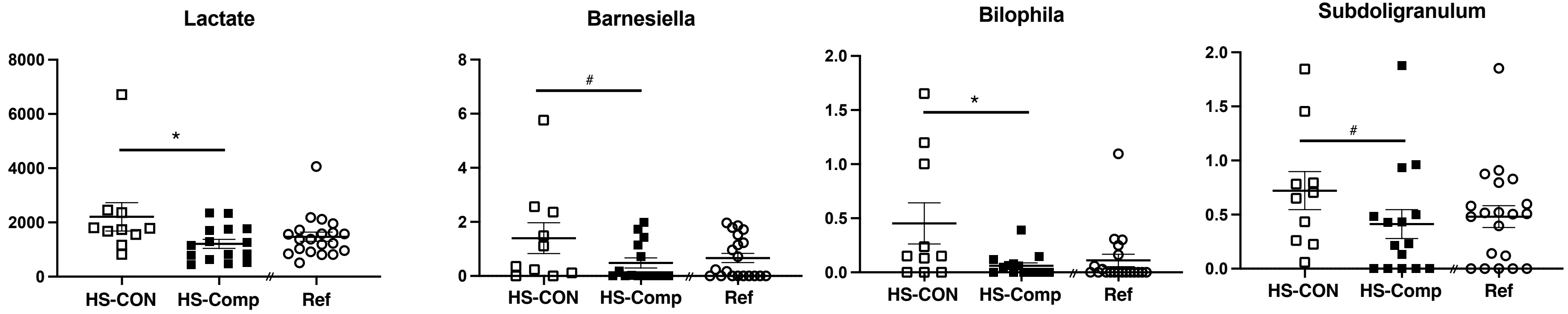

Fig. S8

Relative values of the other EFA components excluding SEM-selected components.

The values of the other EFA components shown in Fig. 5 (excluding the components shown in Fig. 6a) were compared. The marks are shown as follows: *, p<0.05; #, p<0.2.

a

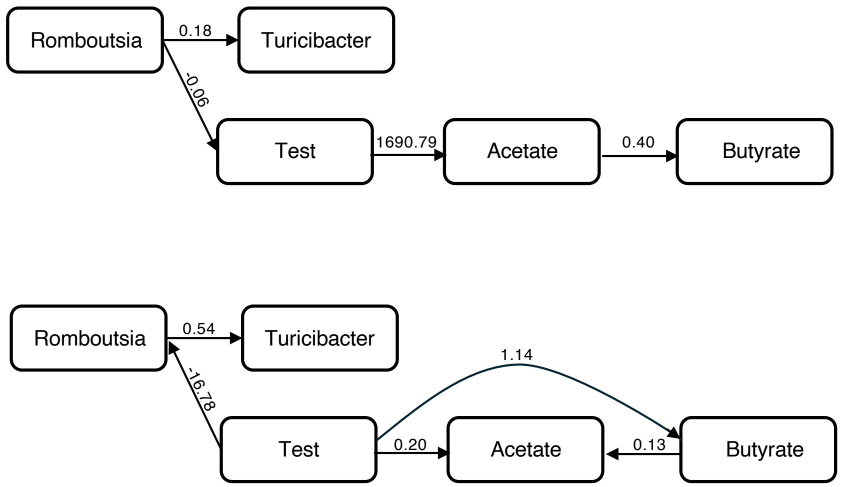

c

Component	X1. Test	X2. Romboutsia	X3. Turicibacter	X4. Acetate	X5. Butyrate
X1. Test	0 (0)	0.0001 (0)	0.129 (0.0005)	0.0001 (0.1712)	0.0453 0.2642)
X2. Romboutsia	0.0001 (0)	0 (0)	0 (0.0137)	0.0002 (0.8315)	0.6714 (0.4043)
X3. Turicibacter	0.129 (0.0005)	0 (0.0137)	0 (0)	0.0047 (0.5679)	0.8117 (0.6793)
X4. Acetate	0.0001 (0.1712)	0.0002 (0.8315)	0.0047 (0.5679)	0 (0)	0.4608 (0.9524)
X5. Butyrate	0.0453 (0.2642)	0.6714 (0.4043)	0.8117 (0.6793)	0.4608 (0.9524)	0 (0)

d

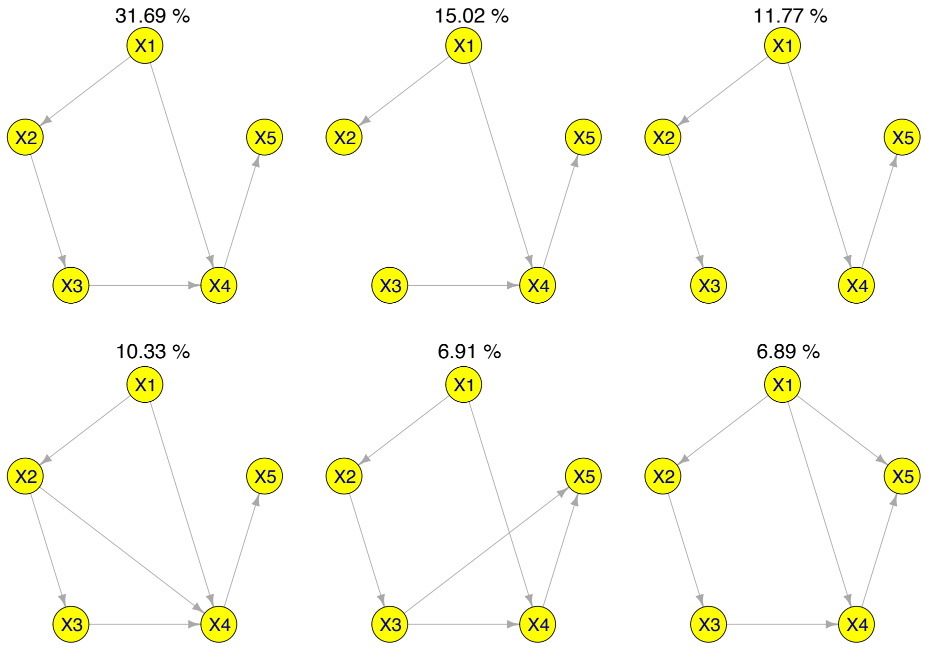

Fig. S9

Validation of the optimal structural equation model by LiNGAM

(a) The directed acyclic graph (DAG) calculated by DirectLiNGAM on the basis of the components for Fig. 5a was visualized. The number on the line represents the contribution value. (b) The DAGs calculated by DirectLiNGAM using CLR-transformed microbial abundance data and log-transformed metabolite. (c) The *p* values calculated by DirectLiNGAM are shown. The numbers in parentheses indicate parameter estimates derived from DirectLiNGAM using CLR-transformed microbial abundance data and log-transformed metabolite data. (d) The DAGs calculated by BayesLiNGAM on the basis of the components for Fig.5c using CLR-transformed microbial abundance data and log-transformed metabolite data standardized by robust z scores. Abbreviations are as follows: X1, Test (the conditions in which the compost extract was administered); X2, Romboutsia; X3, Turicibacter; X4, acetate; X5, butyrate.

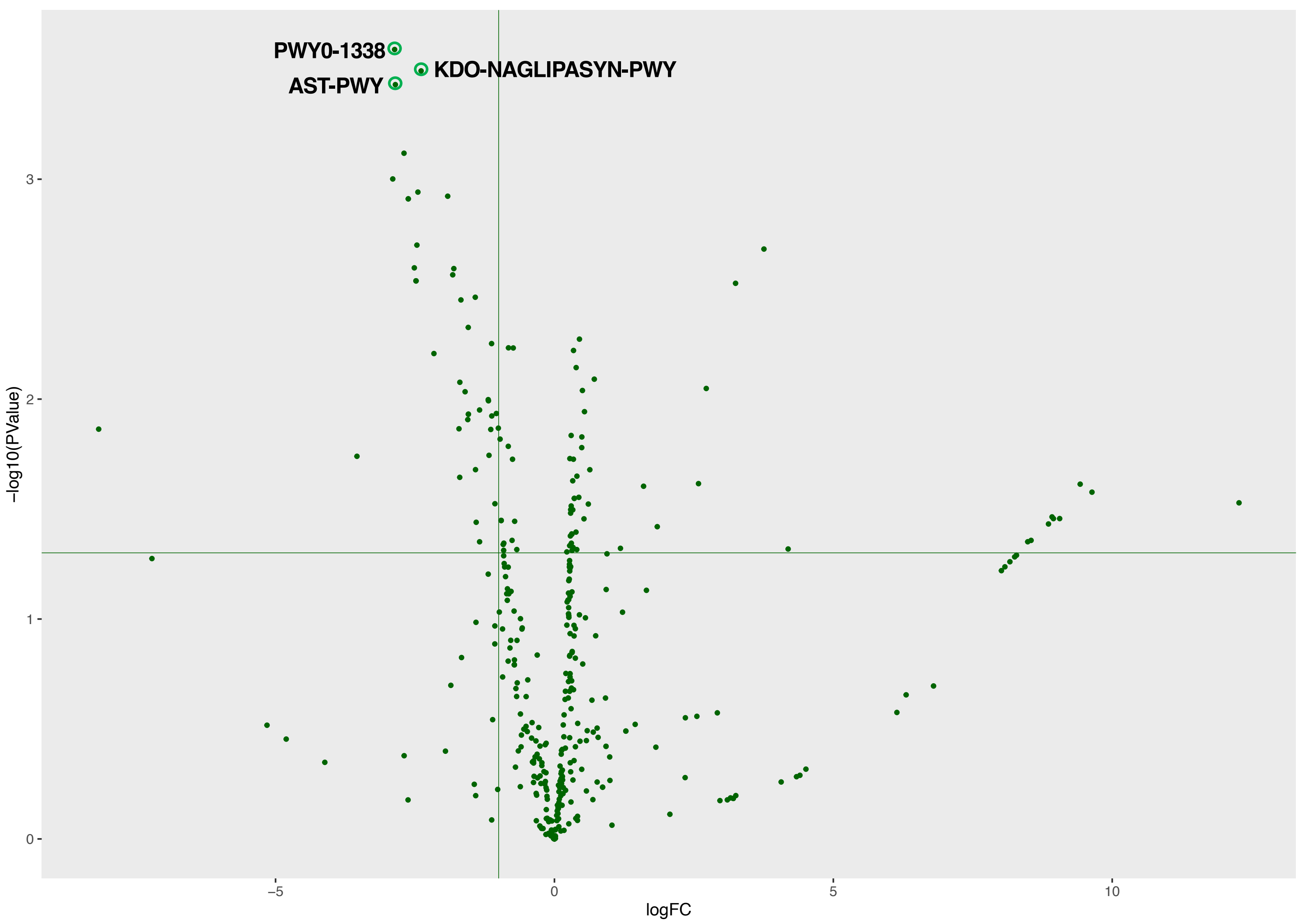

Fig.S10

Volcano plot for pathways calculated at 160 days of age.

The data were classified by false discovery rate (FDR); significant pathways (FDR<0.05) are indicated by the bold letters with green circles.

Table S1
Statistical values for EFA.

Components	Shapiro-Wilk test	ROC curve analysis	EFA score		
		AUC	h2	u2	com
Compost_ex	<0.001	-	0.87	0.1317	3.1
Romboutsia	<0.001	0.0533	1.00	0.0048	1.1
Turicibacter	<0.001	0.1167	1.00	0.0049	1.2
Acetate	5.00E-04	0.9000	0.86	0.1387	1.1
Butyrate	<0.001	0.7667	0.46	0.5418	1
Lactate	<0.001	0.2267	0.70	0.3047	1.5
Subdoligranulum	0.0019	0.2933	0.45	0.5452	1.5
Barnesiella	<0.001	0.2933	0.61	0.3911	2.4
Bilophila	<0.001	0.2533	0.82	0.1802	1.2

The abbreviations in the table are as follows: Compost_ex, a condition in which the compost extract was administered; h2, communality score; u2, uniqueness score; com, complexity, an information score that is generally related to uniqueness; ROC curve analysis, receiver operating characteristic (ROC) curve analysis ; and AUC, area under the curve.

Table S2

Statistical values of the final optimal structural equation models

The values in the category of the optimal model show the fit indices of the best numerical structural equation model in Fig. 6a. The estimator used for these calculations was “MLR” (maximum likelihood robust). The category of the CMA shows the results calculated by the mediation analysis. Numbers in parentheses indicate values derived from structural equation modeling and causal mediation analysis, including parameter estimates and model fit indices. These analyses were performed using CLR-transformed microbial abundance data and log-transformed metabolite data. The abbreviations in the table mean the following: Compost_ex, treatment of the test group with the compost extract; chisq, chi-square χ2; df, degrees of freedom; *p*-value, *p* value from the chi-square test; cfi.robust, robust CFI (comparative fit index); tli.robust, robust TLI (Tucker–Lewis Index); nfi, normed fit index (NFI); rfi, relative fit index (RFI); srmr, standardized root mean residuals (SRMR); AIC, Akaike information criterion; rmsea, root mean square error of approximation (RMSEA); gfi, goodness-of-fit index (GFI); and agfi, adjusted goodness-of-fit index (AGFI); estimate, estimated effect size; Std. Error, standard error; t value, t-statistic (used to test whether the estimate is significantly different from zero); ACME, average causal mediation effect; and ADE, average direct effect.

Category	Contents	Fit indices		
Optimal model	(I) Acetate + Butyrate ~ Compost_ex	chisq 0.137 (6.394)	df 1.000 (4.000)	*p*-value 0.998 (0.172)
	(II) Turicibacter ~ Romboutsia + Compost_ex	cfi.robust 1.000 (0.933)	tli.robust 1.001 (0.849)	rfi 0.996 (0.728)
	(III) Acetate ~ Butyrate	nfi 0.998 (0.879)	srmr 0.010 (0.065)	AIC 912.184 (306.576)
	lavaan 0.6-19 ended normally after 1 iterations	rmsea 0.000 (0.155)	gfi 0.999 (0.891)	agfi 0.976 (0.592)
	Number of successful bootstrap draws: 1000 (1000)			

Category	Contents					
CMA	Nonparametric Bootstrap Confidence Intervals with the Percentile Method					
	Treat: Compost_ex Mediator: Butyrate Outcome: Acetate			Sample Size Used: 25 Simulations: 1000		
		Estimate	95% CI Lower	95% CI Upper	p-value	
	ACME	4.87E+02 (0.764)	1.94E+01 (0.161)	1.42E+03 (1.525)	0.038 (0.012)	* (*)
	ADE	1.20E+03 (1.313)	3.81E+02 (0.659)	2.00E+03 (1.995)	<2e-16 (0.002)	*** (**)
	Total Effect	1.69E+03 (2.077)	1.03E+03 (1.287)	2.33E+03 (2.786)	<2e-16 (<2e-16)	*** (***)
	Prop. Mediated	2.88E-01 (0.368)	1.06E-02 (0.106)	7.66E-01 (0.662)	0.038 (0.012)	* (*)
	Treat: Compost_ex Mediator: Romboutsia Outcome: Acetate			Sample Size Used: 25 Simulations: 1000		
		Estimate	95% CI Lower	95% CI Upper	p-value	
	ACME	23.549 (-0.125)	-223.807 (-1.071)	170.398 (6.002)	0.852 (0.926)	
	ADE	1667.571 (2.202)	957.958 (-3.919)	2323.108 (2.882)	<2e-16 (0.222)	*** (not significant)
	Total Effect	1691.120 (2.077)	1004.211 (1.267)	2348.168 (2.803)	<2e-16 (<2e-16)	*** (***)
	Prop. Mediated	0.014 (-0.060)	-0.151 (-0.684)	0.105 (3.103)	0.852 (0.926)	
